# Persistent cAMP-Signals Triggered by Internalized G-Protein–Coupled Receptors

**DOI:** 10.1371/journal.pbio.1000172

**Published:** 2009-08-18

**Authors:** Davide Calebiro, Viacheslav O. Nikolaev, Maria Cristina Gagliani, Tiziana de Filippis, Christian Dees, Carlo Tacchetti, Luca Persani, Martin J. Lohse

**Affiliations:** 1 Institute of Pharmacology and Toxicology, University of Würzburg, Würzburg, Germany; 2 Rudolf Virchow Center, DFG-Research Center for Experimental Biomedicine, University of Würzburg, Würzburg, Germany; 3 Dipartimento di Scienze Mediche, Università degli Studi di Milano, Milan, Italy; 4 Laboratory of Experimental Endocrinology, Fondazione IRCSS Istituto Auxologico Italiano, Cusano Milanino, Italy; 5 Department of Experimental Medicine, University of Genoa, Genoa, Italy; University of California San Francisco, United States of America

## Abstract

G-protein–coupled receptors (GPCRs) are generally thought to signal to second messengers like cyclic AMP (cAMP) from the cell surface and to become internalized upon repeated or prolonged stimulation. Once internalized, they are supposed to stop signaling to second messengers but may trigger nonclassical signals such as mitogen-activated protein kinase (MAPK) activation. Here, we show that a GPCR continues to stimulate cAMP production in a sustained manner after internalization. We generated transgenic mice with ubiquitous expression of a fluorescent sensor for cAMP and studied cAMP responses to thyroid-stimulating hormone (TSH) in native, 3-D thyroid follicles isolated from these mice. TSH stimulation caused internalization of the TSH receptors into a pre-Golgi compartment in close association with G-protein α_s_-subunits and adenylyl cyclase III. Receptors internalized together with TSH and produced downstream cellular responses that were distinct from those triggered by cell surface receptors. These data suggest that classical paradigms of GPCR signaling may need revision, as they indicate that cAMP signaling by GPCRs may occur both at the cell surface and from intracellular sites, but with different consequences for the cell.

## Abstract 2

Real-time monitoring of G-protein-coupled receptor (GPCR) signaling in native cells suggests that the receptor for thyroid stimulating hormone remains active after internalization, challenging the current model for GPCR signaling.

## Author Summary

Cells respond to many environmental cues through the activity of cell surface receptor proteins, which sense these cues and convey that information to signaling molecules inside the cell. G-protein–coupled receptors (GPCRs) form the largest eukaryotic family of plasma membrane receptors. They convert the information provided by extracellular stimuli into intracellular second messengers, like cyclic AMP (cAMP). After prolonged stimulation, they are internalized inside cells, an event that to date has been thought to terminate the production of second messengers. Though many of the key steps of GPCR signaling are known in detail, precisely how signaling and termination actually occur in time and space (i.e., in subcellular compartments or microdomains) is still largely unexplored. To observe GPCR signaling in living cells, we generated mice expressing a fluorescent sensor that allows monitoring the intracellular levels of cAMP with a microscope. We utilized this system to study, directly in native thyroid follicles, the signal sent by the receptor for thyroid-stimulating hormone (TSH). Our findings indicate that TSH receptors are internalized rapidly after activation but continue to stimulate cAMP production inside cells and that this sustained, cAMP production is apparently required for localized activation of downstream components. These data challenge the current model of the GPCR-cAMP pathway by suggesting the existence of previously unrecognized intracellular site(s) for cAMP generation and of differential signaling outcomes as a result of intracellular GPCR signaling. Such intracellular site(s) may provide specialized signaling platforms, thus contributing to the spatiotemporal regulation of cAMP production and to signaling specificity within the GPCR family.

## Introduction

G-protein–coupled receptor (GPCR) signaling is thought to involve a series of steps occurring at the cell surface: coupling of receptors to G-proteins, activation of G-proteins, and ultimately, triggering of G-protein-regulated effectors (i.e., adenylyl cyclase, phospholipase C, calcium channels, GIRK channels, etc.) [1]. Soon after activation, many GPCRs desensitize in a process that involves phosphorylation by G-protein–coupled receptor kinases (GRKs) and binding of β-arrestins [1]. Subsequently, most GPCRs are internalized via clathrin-coated pits or other less characterized pathways, and are either dephosphorylated and recycled back to the cell surface or targeted to lysosomes for degradation [1].

Although receptor internalization was originally considered to contribute to desensitization by reducing the number of receptors present on the cell plasma membrane, endocytosis has been subsequently and unexpectedly found to promote or even be required for receptor resensitization [1],[2]. Furthermore, novel data suggest that receptor internalization does not always lead to signal termination. This possibility has been clearly demonstrated for tyrosine kinase receptors, such as the epidermal growth factor receptor (EGFR), that were shown to continue signaling after being internalized [3]–[6]. In the case of GPCRs, instead, internalized receptors are thought capable of switching to a “nonconventional” signaling pathway, i.e., a β-arrestin-mediated activation of the mitogen-activated protein kinase (MAPK) cascade [7]. A very recent study has revealed yet another type of intracellular GPCR signaling in yeast: Gpa1, the yeast homolog of Gα can be activated by pheromone receptors on endosomes, where it stimulates phosphatidylinositol 3-phosphate production [8]. Despite such recent data, there is a current consensus that activation of canonical G-protein effectors, such as adenylyl cyclase, by GPCRs occurs exclusively at the cell surface.

Describing the spatiotemporal dynamics of signaling cascades is a major goal of cell biology. In the case of GPCR signaling, this would imply answering fundamental questions such as: Where in the cell and for how long are GPCRs active after interacting with their ligands? Are there subcellular microdomains specialized for different types of GPCR-mediated signals? What are the functional consequences of GPCR internalization on signaling? Does signaling to cyclic AMP (cAMP) or other second messengers occur only at the plasma membrane, or are there additional sites of GPCR activity inside the cell? Most of these questions are still awaiting answers. The major reason for this relies on the fact that biochemical techniques, until recently the only ones available for such analyses, require cell disruption and therefore have limited temporal and, generally, no spatial resolution. To tackle those limitations, we and others have developed a series of genetically encoded fluorescent reporters that allow the direct visualization of key steps of GPCR [9]–[11] and cyclic nucleotide signaling [12]–[19], by means of microscopy techniques based on fluorescence resonance energy transfer (FRET). The introduction of these techniques has led to new insights into the mechanisms of GPCR activation and the biology of cAMP.

Despite the important advance represented by the introduction of FRET-based techniques, most studies required transfection of genetically encoded fluorescent reporters into primary cells or cell lines—thus, quite far from the physiological context. This is a fundamental issue, as the type, location, and concentration of each component, as well as the size and shape of cells, are expected to greatly influence the spatiotemporal features of signaling networks [20]–[22]. To be able to study GPCR-cAMP signaling in a highly physiological context, here we generated reporter mice with ubiquitous expression of an inert fluorescent sensor for cAMP. These mice were utilized to study the dynamics of a GPCR-cAMP signaling cascade, i.e., that activated by thyroid-stimulating hormone (TSH), within the intact multicellular functional unit that constitutes thyroid tissue.

## Results

### Generation of Transgenic Mice with Ubiquitous Expression of a cAMP Sensor

**Figure 1:**
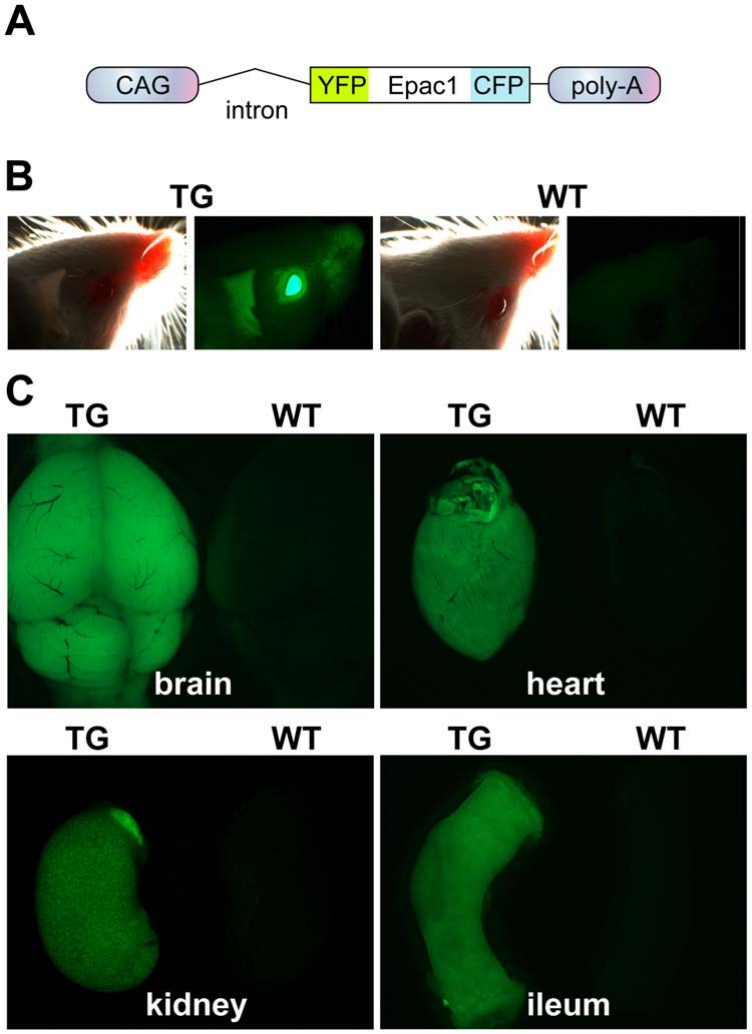
Generation and characterization of CAG-Epac1-camps transgenic mice. (A) Expression cassette used to generate the transgenic mice. The cAMP sensor (Epac1-camps) contains a cAMP binding domain derived from Epac1, flanked on either side by YFP and CFP. The Epac1-camps sensor is under the control of the ubiquitous CAG promoter. (B) Fluorescent image of the head of a transgenic (TG) mouse compared to that of a wild-type (WT) littermate. (C) Fluorescent images of different organs isolated from adult TG and WT mice.

To monitor cAMP levels in living cells and tissues, we generated transgenic mice (CAG-Epac1-camps) with ubiquitous expression of our previously described cAMP sensor (Epac1-camps) [14]. We followed the same strategy used to create green fluorescent protein (GFP) mice [23]. Instead of GFP, we cloned the Epac1-camps sequence (encompassing the yellow fluorescent protein [YFP], the cAMP binding domain of Epac1, and the cyan fluorescent protein [CFP]) under the control of the hybrid CMV enhancer/chicken β-actin (CAG) promoter (Figure 1A), and performed pronuclear injections of one-cell–stage mouse embryos with this construct. After several rounds of injections, we obtained ten PCR-positive pups, three of which showed a high level of body fluorescence and gave rise to transgenic offspring according to the Mendelian ratio. Careful analysis of isolated cells and tissues from these three lines demonstrated that two of them had a heterogeneous expression of the sensor, which was present in only 60%–80% of the cells. By contrast, the third line expressed Epac1-camps in virtually all cells and was therefore chosen for subsequent experiments. Figure 1 reproduces fluorescent images of the head and a series of organs isolated from the transgenic mice. Compared to wild-type littermates, these mice had high levels of sensor expression in almost all tissues and cells, excluding erythrocytes and hair. For example, we found high fluorescence in the eye and the skin (Figure 1B), as well as in the brain, heart, kidney, and ileum (Figure 1C). Interestingly, transgenic mice did not show any abnormalities and had a normal life expectancy, demonstrating that the presence of the sensor in most cells of the organism did not interfere with its proper development and physiological functions.

### Real-Time cAMP Measurements

**Figure 2:**
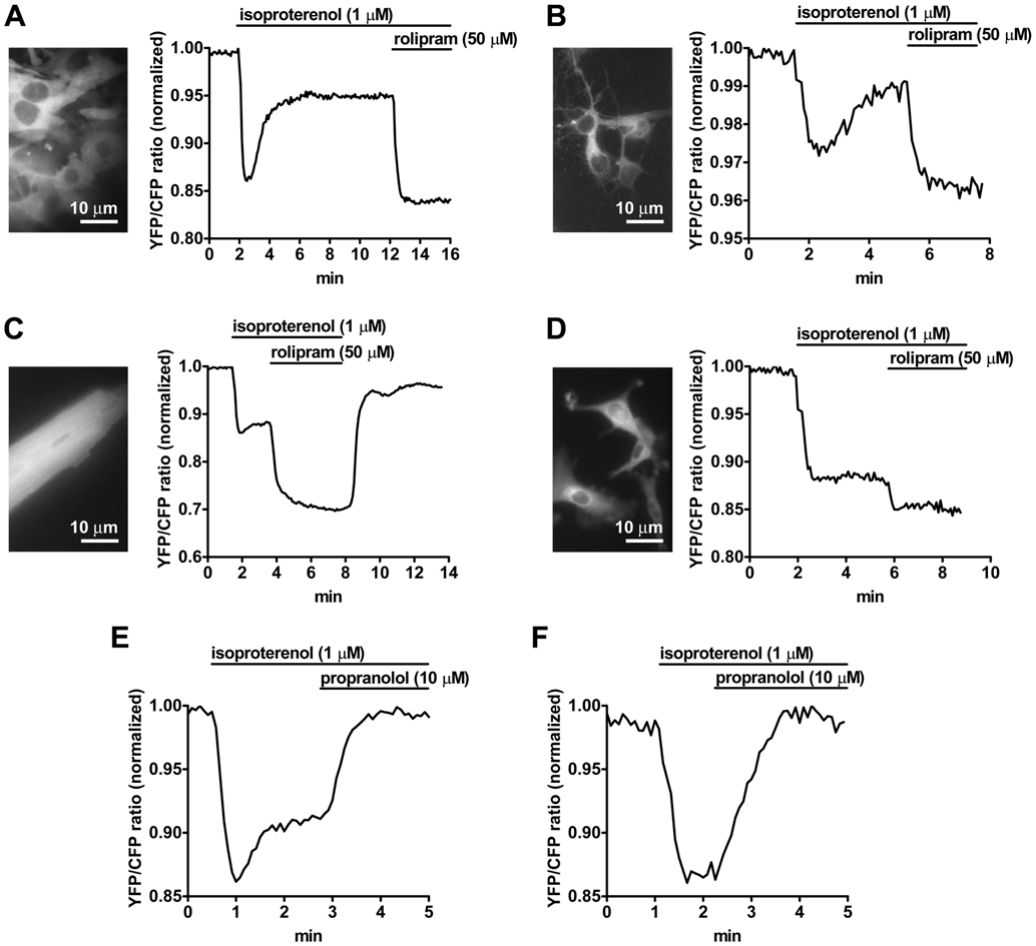
Real-time monitoring of cAMP levels in different types of primary cells isolated from the cAMP reporter mice. Cells were visualized by time-lapse fluorescence microscopy. The graphs show normalized YFP/CFP ratio values calculated from CFP and YFP images. A reduction of the YFP/CFP ratio is indicative of an increase of cAMP levels. (A) Murine embryonic fibroblasts (MEFs) were stimulated with the β-adrenergic agonist isoproterenol. The cAMP response to isoproterenol was robust but transient due to the activation of PDE4, as indicated by the strong effect of the PDE4-selective inhibitor rolipram. (B) Cortical neurons reacted to isoproterenol and rolipram in a similar way. (C) The response to β-adrenergic stimulation of cardiac cells was further enhanced by rolipram. (D) Peritoneal macrophages showed a more sustained increase in cAMP levels after isoproterenol stimulation and a minor effect of rolipram. (E and F) The isoproterenol effect on cardiac myocytes (E) and macrophages (F) was completely blocked by the β-adrenergic antagonist propranolol. Traces in (A–F) are representative of three to ten experiments per condition.

Next, we isolated several types of embryonic and adult primary cells from CAG-Epac1-camps mice to measure cAMP levels in real time. cAMP levels were monitored by FRET microscopy on live cells as previously described [14]. From E14.5 embryos, we obtained murine embryonic fibroblasts (MEFs) and cortical neurons, which showed high fluorescence. Stimulation of MEFs with the β-adrenergic agonist isoproterenol resulted in a robust decrease of the YFP/CFP ratio (Figure 2A), indicative of an increase of cAMP levels [14]. As already reported in other cell types [24],[25], this response was transient, due to the protein kinase A (PKA)-dependent activation of phosphodiesterase 4 (PDE4), which could be counteracted by addition of a specific inhibitor (rolipram). In cortical neurons, isoproterenol induced a similar type of reaction, though of smaller amplitude. Like in MEFs, the response was transient and could be enhanced by addition of rolipram (Figure 2B). From adult mice, we isolated cardiac myocytes and peritoneal macrophages. In line with our previous observations [26], β-adrenergic stimulation of cardiac cells led to an increase of cAMP levels, which was further enhanced by rolipram (Figure 2C). The cAMP response to isoproterenol in macrophages was more sustained and only minimally affected by rolipram (Figure 2D). Finally, the β-adrenergic antagonist propranolol completely inhibited the effect of isoproterenol on cAMP production in cardiomyocytes (Figure 2E) and macrophages (Figure 2F), thus showing that the observed FRET variations were specific.

### Establishment of a 3-D Culture of Thyroid Follicles

**Figure 3:**
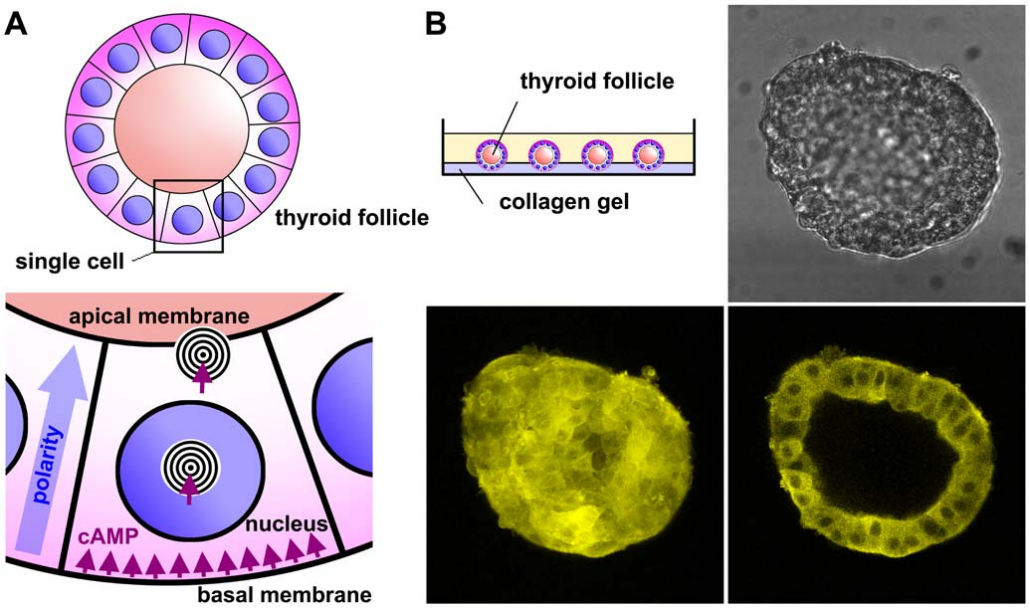
The thyroid follicle model. (A) Thyroid follicles constitute the anatomical and functional units of thyroid tissue. They are composed of a monolayer of epithelial cells that defines an inner cavity where thyroid hormones are stored in the form of an iodinated protein (thyroglobulin). Upon binding of TSH to its receptor located on the basolateral membrane, cAMP is produced with consequent activation of PKA and phosphorylation of a series of targets, located in different cellular compartments (e.g., cytosol, nucleus, Golgi complex, apical membrane). These events lead to a fast induction of thyroglobulin reuptake with release of free thyroid hormones into the blood stream and a slow up-regulation of thyroglobulin synthesis and iodination. Thyroid cells are highly polarized, as the basolateral and apical membranes have completely different compositions and extremely specialized functions. (B) Method used to culture thyroid follicles. Isolated mouse thyroid follicles were placed in a glass-bottom Petri dish, coated with a thin layer of collagen gel. Shown are representative images of a single follicle isolated from CAG-Epac1-camps mice after 12 h of culture. Top right, bright field image. Bottom left, maximum projection of YFP fluorescence (corresponding to the Epac1-camps sensor) calculated from individual image slices on the *z*-axis, captured with a laser-scanning confocal microscope. Bottom right, single confocal image of YFP fluorescence.

Then, we utilized CAG-Epac1-camps mice to monitor GPCR-cAMP signaling in an intact physiological system. We chose thyroid cells for several reasons. First, these cells are strictly dependent for all their functions, e.g., thyroid hormone production and growth, on the activation of a GPCR, the TSH receptor, which is expressed on their basolateral membrane and the effects of which are largely mediated by cAMP [27],[28]. Second, thyroid cells form supracellular structures, known as thyroid follicles, which constitute both the anatomical and the functional units of thyroid tissue. Importantly, it is possible to isolate and culture thyroid follicles so to maintain their original 3-D structure and polarization, which is reflected by a high spatial organization of the TSH receptor-cAMP signaling cascade [27],[29],[30] (Figure 3A). Thus, they represent a unique model to study the spatiotemporal dynamics of GPCR-cAMP signaling. For this purpose, we established a method that allowed us to isolate, maintain, visualize, and manipulate mouse thyroid follicles under the microscope. Among various protocols, the best results were obtained by enzymatic dissociation followed by deposition on a thin layer of collagen gel (Figure 3B). This protocol preserved good 3-D morphology for at least 24–48 h of culture, without hampering microscopic observation or manipulation (Figure 3B and Video S1).

### Real-Time Monitoring of cAMP Levels in Intact Thyroid Follicles

**Figure 4:**
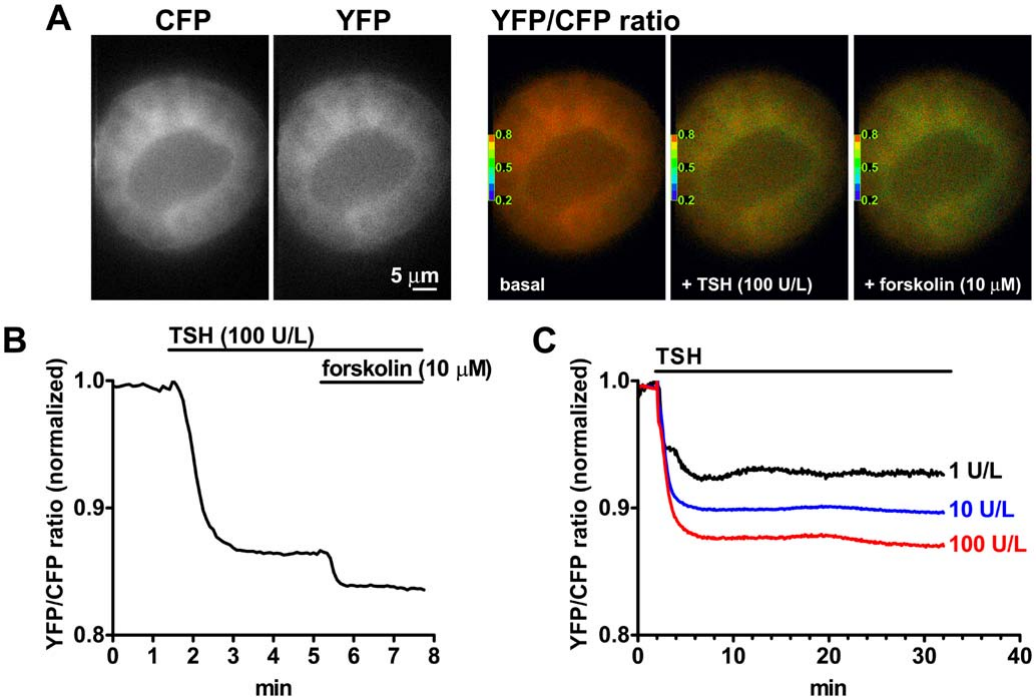
Real-time monitoring of cAMP levels in thyroid follicles. Thyroid follicles isolated from CAG-Epac1-camps mice were visualized by time-lapse fluorescence microscopy. (A) CFP, corrected YFP and YFP/CFP ratio images from a representative sequence, where a thyroid follicle was stimulated with TSH followed by forskolin. (B) Normalized YFP/CFP ratio values obtained from the sequence in (A). (C) Effect of prolonged stimulation with different concentrations of TSH on intracellular cAMP levels. Results in (A–C) are representative of five to ten experiments per condition.

Next, we utilized thyroid follicles isolated from CAG-Epac1-camps mice to monitor in real time the cAMP response to TSH stimulation. Saturating concentrations of TSH resulted in a fast and robust decrease of the YFP/CFP ratio (Figure 4A and 4B and Video S2), indicative of an increase of cAMP levels [14]. Nevertheless, the Epac1-camps sensor was not saturated, as shown by the further decrease of FRET values obtained by fully activating adenylyl cyclase with forskolin. In contrast to what was observed in other cell types, like for example, embryonic fibroblasts and cortical neurons (for comparison see Figure 2A and 2B), no reduction of cAMP levels was seen after prolonged exposure to various concentrations of TSH (up to 30 min), consistent with limited or no PDE activation under our experimental conditions (Figure 4C). This type of sustained cAMP response further drove our attention to TSH receptor signaling in thyroid cells.

To rule out the possibility that the presence of the Epac1-camps sensor might interfere with cAMP signaling, we compared the cAMP-response to TSH stimulation in thyroid cells isolated from wild-type and CAG-Epac1-camps mice. The intracellular levels of cAMP, measured by an immunoenzymatic assay, were indistinguishable between wild-type and transgenic cells (Figure S1).

### Temporal Dynamics of cAMP Signaling in Thyroid Follicles

**Figure 5:**
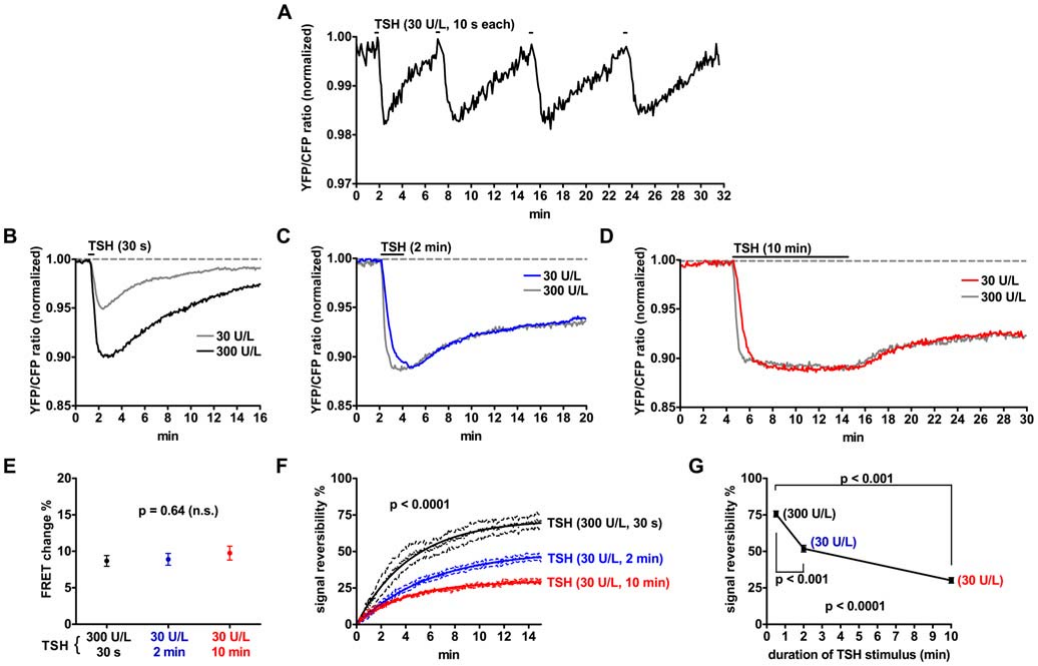
Effect of transient TSH stimulation on cAMP levels. Thyroid follicles isolated from CAG-Epac1-camps mice were visualized by time-lapse fluorescence microscopy. (A) Effect of repeated short stimuli (10 s each) with TSH. (B–D) Effect of longer TSH applications. Reported are data from representative experiments in which thyroid follicles were stimulated for 30 s (B), 2 min (C), or 10 min (D). (E) Mean FRET changes induced by stimuli as in (B–D). Values were compared by one-way ANOVA. (F) Comparison of signal recovery after stimuli as in (B–D). Signal reversibility was calculated from the YFP/CFP ratio data of the washout phase, by setting the value at the end of TSH stimulation equal to zero and the value before TSH stimulation equal to 100%. The values obtained from different replicates were globally fitted to a first-order exponential function. Fits were compared with F test, having a null hypothesis that *Y*_max_ values were the same for all datasets. (G) Comparison of *Y*_max_ values obtained from fitting each dataset in (F) to a first-order exponential equation. Values were compared by one-way ANOVA, followed by Bonferroni post hoc test. Data in (E–G) were obtained from six to eight independent experiments per condition.

Importantly, the lack of appreciable desensitization of the TSH receptor-cAMP signal allowed us to evaluate the kinetics of the return of cAMP to baseline after transient stimulation with TSH. To ensure controlled and fast stimulation, thyroid follicles were kept under laminar-flow perfusion with an apparatus that allowed the rapid exchange between different extracellular solutions. First, we applied very short (10 s) and repeated stimuli with TSH (30 U/l), which were followed by a complete return of cAMP to basal values (Figure 5A). Then, we stimulated thyroid follicles with saturating (30 U/l) or supra-saturating (300 U/l) concentrations of TSH for 30 s (Figure 5B), 2 min (Figure 5C), or 10 min (Figure 5D). Note that 300 U/l were required to reach maximum activation within 30 s, whereas 30 U/l were sufficient for 2-min and 10-min applications. Stimulation with these fully activating concentrations of TSH produced comparable changes of cAMP levels, independently of the duration of the application (Figure 5E). Unexpectedly, stimuli of duration equal or longer than 30 s were associated with an incomplete return of cAMP to basal levels even after extensive washout (Figure 5B–5D). Interestingly, the extent of signal irreversibility increased with the duration of the TSH application, but was not dependent on the cumulative dose of TSH (for example, compare the effect of 300 U/l for 30 s to that of 30 U/l for 2 min), occurred already after 30 s, and was nearly maximal after 10-min stimulation (Figure 5F and 5G). In contrast to TSH receptor activation, stimuli of comparable intensity and duration with a forskolin analog, which directly activates adenylyl cyclase, yielded completely reversible cAMP signals (Figure S2).

**Figure 6:**
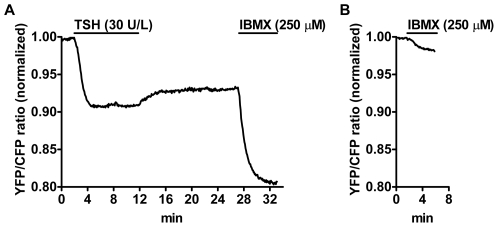
Role of PDEs on cAMP signal irreversibility. (A) Thyroid follicles isolated from CAG-Epac1-camps mice were stimulated with TSH for 10 min followed by extensive washout. Thereafter, a nonselective PDE inhibitor (IBMX) was added to probe the PDE activity. (B) For comparison, the effect of IBMX was evaluated on thyroid follicles that were not previously stimulated with TSH. Shown are representative traces from four to six experiments per condition.

In theory, the incomplete restoration of cAMP levels observed after prolonged TSH stimulation might have been due to an inactivation of PDEs. To investigate this possibility, we added a nonselective PDE inhibitor (IBMX) at the end of the washout phase. In contrast to control follicles that were not previously stimulated with TSH, on which IBMX had only a marginal effect, IBMX treatment caused a robust increase in cAMP levels when applied after TSH stimulation for 10 min and subsequent washout (Figure 6). These results demonstrated that, after extensive washout from a prolonged TSH stimulus, PDEs were still highly functional. Thus, it is unlikely that an inactivation of PDEs was the cause of the incomplete recovery of cAMP levels. On the contrary, the fast and robust increase of cAMP levels after IBMX addition suggested that the receptor-adenylyl cyclase system was indeed continuing to signal.

### TSH Receptor Internalization

The extent and kinetics of endogenous TSH receptor internalization in primary thyroid cells have been difficult to evaluate due to its very low expression levels. However, the available information suggests that the TSH receptor is internalized and recycled back to the plasma membrane, without being targeted to lysosomes [31],[32]. To monitor the internalization of the endogenous TSH receptor, we utilized the well-established method of following the endocytosis of a fluorescent ligand [3]. For this purpose, we labeled bovine TSH with a fluorescent dye (Alexafluor594), which resulted in no major alterations of its biological activity (Figure S3A); in addition, TSH-Alexa594 bound specifically to HEK293 cells transfected with TSH receptor cDNA (Figure S3B).

**Figure 7:**
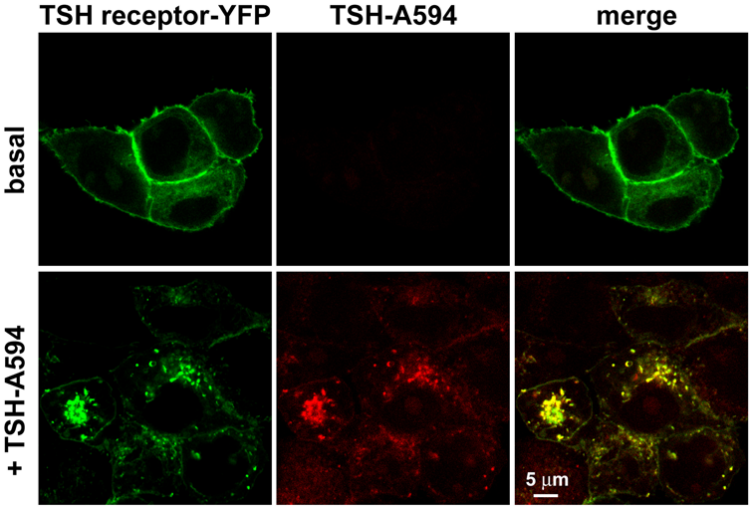
Cointernalization of TSH and its receptor in HEK293 cells. HEK293 cells transfected with YFP-tagged TSH receptor and β-arrestin 2 were stimulated with 3 µg/ml TSH-Alexa594 for 40 min, fixed, and then visualized by confocal microscopy. “Basal” refers to control cells that were not stimulated with TSH-Alexa594. Images are representative of three independent experiments.

Initially, to simultaneously monitor TSH and TSH receptor internalization, we cotransfected HEK293 cells with a YFP-tagged TSH receptor construct and β-arrestin 2, which in this context is required for efficient TSH receptor internalization [33], and stimulated them with TSH-Alexa594 for different periods of time. Both ligand and receptor were found to cointernalize rapidly, reaching a maximum after approximately 20 min (unpublished data). Interestingly, 40 min after stimulation, the ligand and the receptor still appeared to be present in the same intracellular compartments, suggesting that they were not sorted apart during this period of time (Figure 7).

**Figure 8:**
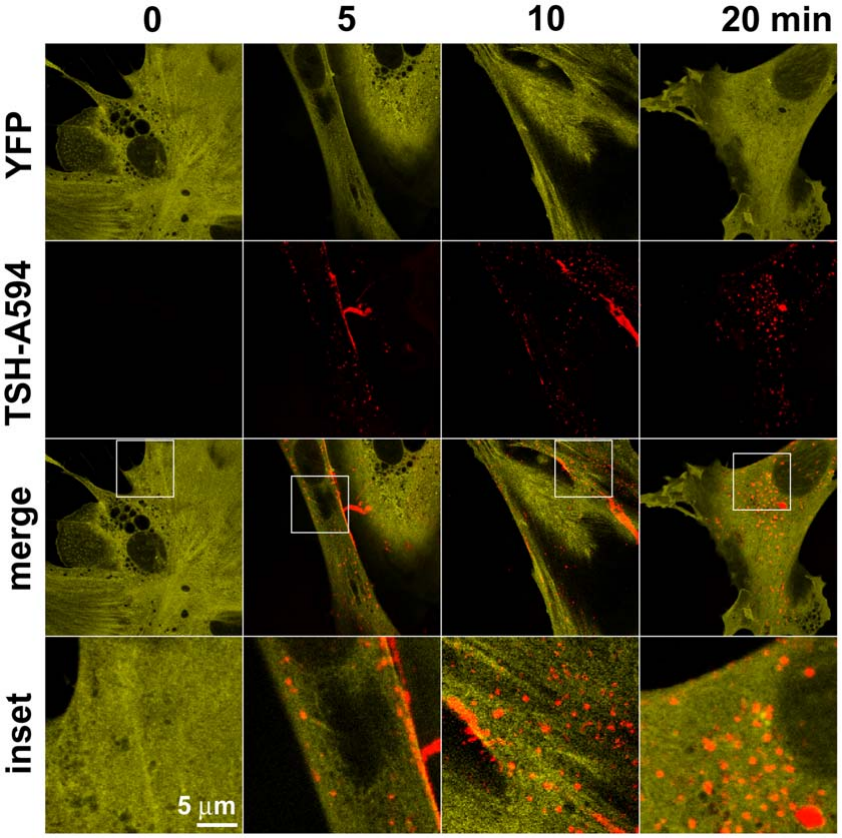
Time-course analysis of TSH receptor internalization. Primary thyroid cells obtained from CAG-Epac1-camps mice were stimulated with 3 µg/ml TSH-Alexa594 for the indicated period of time, fixed, and then visualized by confocal microscopy. YFP images of Epac1-camps were used as a cytosolic counterstain. Images are representative of 25–30 cells per condition analyzed in four independent experiments.

**Figure 9:**
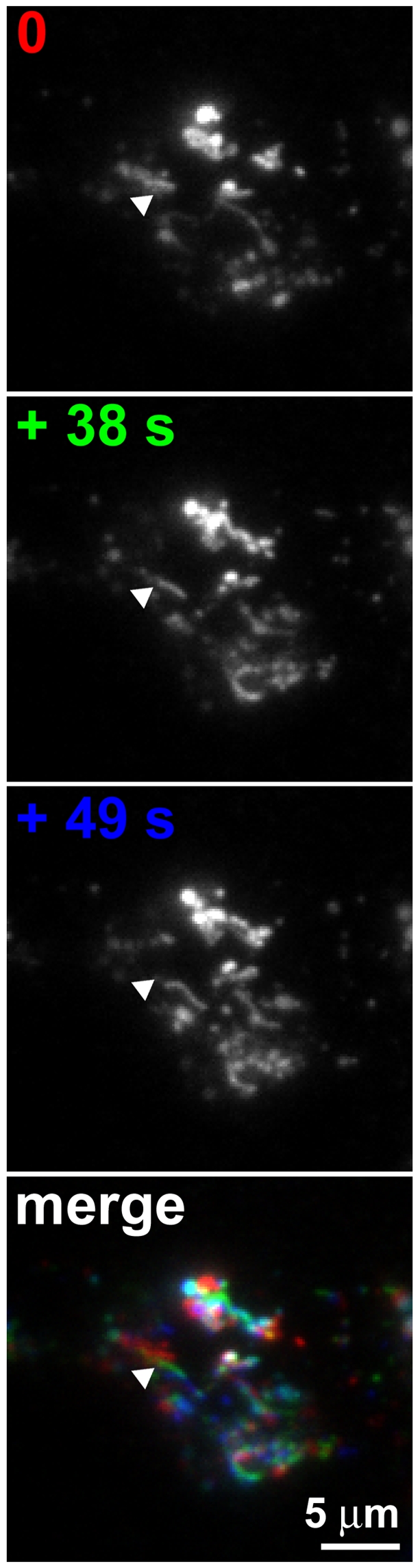
Dynamic visualization of internalized TSH-Alexa594. Primary mouse thyroid cells were stimulated with 3 µg/ml TSH-Alexa594 for 20 min. Thereafter, the TSH-Alexa594 fluorescence was visualized with a TIRF microscope set to have a high penetration depth. Shown are three representative frames, acquired at the indicated time points. The arrowhead indicates a tubule that extended during the observation. The merged image was produced by overlaying the images of the three individual frames, after coloring them in red (0 s), green (+38 s) and blue (+49 s). White indicates regions of the image that did not change during this period of time. Data are representative of 20 sequences from four independent experiments.

Then, we utilized the fluorescent ligand to follow TSH receptor internalization in primary thyroid cells. When we attempted to visualize whole thyroid follicles loaded with TSH-Alexa594, we found them to have a relatively high autofluorescence in the whole visible spectrum. Thus, visualization of TSH-Alexa594 was largely hampered, and possible only to a limited extent through spectral unmixing. The resulting images, though of poor quality, were suggestive of TSH-Alexa594 being efficiently internalized in thyroid follicles (Figure S4). To better evaluate this phenomenon, we analyzed the binding and internalization of TSH-Alexa594 in single primary mouse thyroid cells, which showed much lower autofluorescence. Interestingly, we found that TSH internalized quickly, with TSH-Alexa594–positive vesicles being detectable 5 min after TSH stimulation and maximal internalization reached approximately after 20 min (Figure 8). Importantly, at this point, a major fraction of labeled TSH was found inside the cells, in vesicles prevalently concentrated around the nucleus. To further investigate the structure and the dynamics of TSH-containing vesicles, we performed a series of time-lapse experiments on cells stimulated with TSH-Alexa594 that were visualized with a total internal reflection fluorescence (TIRF) microscope set to have a high penetration depth. This approach allows us to visualize cytoplasmic structures close to the plasma membrane with a high signal/background ratio [34] and appeared particularly suited for thyroid cells that assume a very thin shape in culture, having a maximal thickness of approximately 1–2 µm only (unpublished data). Interestingly, we found that TSH-Alexa594 was contained in tubulovesicular organelles, forming a highly dynamic and interconnected network (Figure 9 and Video S3), the structure of which could be appreciated only partially by confocal microscopy.

### Subcellular Localization of G-Proteins and Adenylyl Cyclases

So far, our results suggested that the TSH receptor internalized quickly without evidence of cAMP desensitization and that, after prolonged stimulation, a significant proportion of the TSH receptor-cAMP signal became irreversible. Of note, both phenomena, i.e., receptor internalization and signal irreversibility, had roughly similar kinetics. We therefore hypothesized that, over time, a fraction of the receptors was brought to a state or a compartment where they could no longer be freed of their ligands, but were still able to signal. Trimeric G-proteins are frequently found at non-plasma membrane compartments, such as endosomes, the endoplasmic reticulum, and the Golgi, where they are thought to play roles in vesicle trafficking [8],[35],[36]. Similarly, adenylyl cyclase activity and immunoreactivity have been found on intracellular membranes, though such studies were frequently limited by the very low expression of adenylyl cyclases and the relatively low affinity of the available antibodies [37],[38]. Therefore, it appeared possible that the other components required for TSH receptor signaling might also be present inside the cell. Based on these considerations, we attempted to localize Gα_s_ as well as adenylyl cyclase III, V, and VI, the major isoforms expressed in thyroid cells [39], by immunofluorescence. Our results indicated that Gα_s_ was present on intracellular vesicles and tubulovesicular structures that were typically concentrated around the nucleus (Figures S5, 10, and 11A and 11B). Adenylyl cyclase staining was of much lower intensity. Nonetheless, adenylyl cyclase immunoreactivity was found occasionally on the plasma membrane and largely on intracellular vesicles (Figures S5 and 11C). TSH stimulation did not cause apparent modifications of Gα_s_ or adenylyl cyclase subcellular localization (unpublished data). The specificity of Gα_s_, adenylyl cyclase III, and adenylyl cyclase V/VI staining was checked by competition with the peptides used to raise the primary antibodies (Figure S6). In addition, the specificity of Gα_s_ immunofluorescence was evaluated by comparing its subcellular pattern to the localization of YFP-tagged Gα_s_ transfected in HEK293 cells (unpublished data).

**Figure 10:**
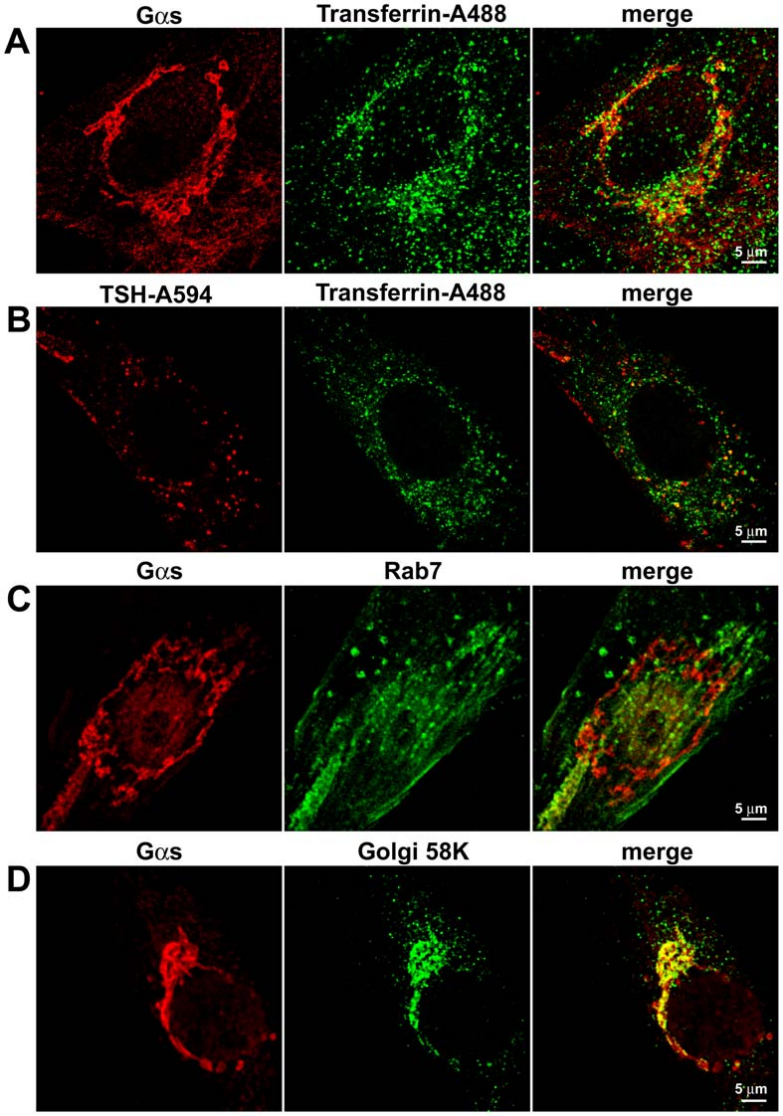
Colocalization between Gα_s_ and subcellular markers. (A) Colocalization between Gα_s_ and Alexafluor488-conjugated transferrin (transferrin-Alexa488), used to visualize early and recycling endosomes. Primary mouse thyroid cells were stimulated for various periods of time (2–60 min) with transferrin-Alexa488, followed by immunofluorescence analysis for Gα_s_. No colocalization was observed at early time points (2–5 min) (unpublished data). At later time points (20–60 min), transferrin appeared to be contained in vesicles associated with the perinuclear tubulovesicular structure positive for Gα_s_. Some of these vesicles were also positive for Gα_s_. Reported is a representative image of a cell treated with transferrin-Alexa488 for 20 min. (B) Colocalization between TSH-Alexa594 and transferrin-Alexa488, in a cell that was simultaneously treated with both fluorescent ligands for 20 min. A partial colocalization between TSH-Alexa594 and transferrin-Alexa488 was observed. (C) Colocalization between Gα_s_ and Rab7, used as a marker of late endosomes. Cells were analyzed by double-immunofluorescence with antibodies against Gα_s_ and Rab7. No colocalization was observed. (D) Colocalization between Gα_s_ and Golgi 58K, used as a marker for the Golgi complex. Cells were analyzed by double-immunofluorescence with antibodies against Gα_s_ and Golgi 58K. A high degree of colocalization was observed. Images in (A–D) are representative of more than 20 cells per condition analyzed in at least three independent experiments.

**Figure 11:**
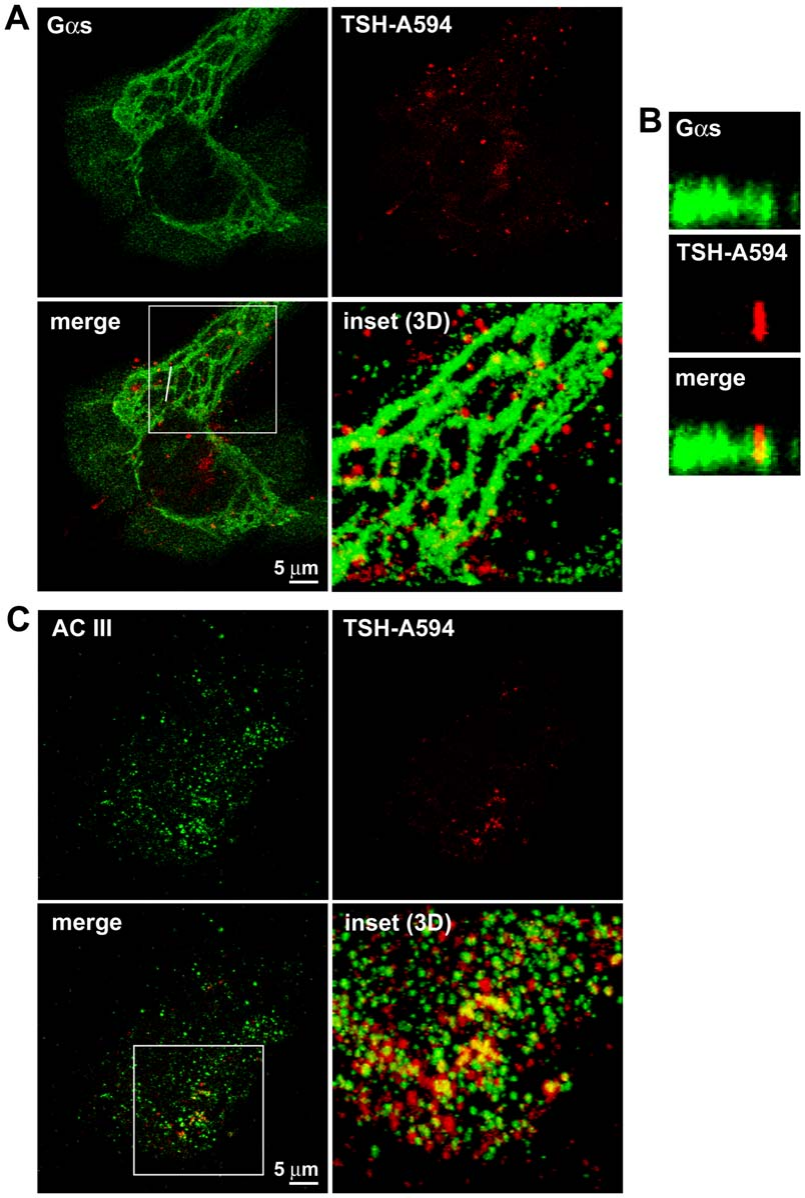
Subcellular localization of Gα_s_, adenylyl cyclase III, and internalized TSH in primary thyroid cells. Primary mouse thyroid cells were stimulated with 3 µg/ml TSH-Alexa594 for 10 min, followed by immunofluorescence analysis with antibodies against Gα_s_ (A and B) or adenylyl cyclase III (C). Image stacks on the *z*-axis were acquired with a laser-scanning confocal microscope. Shown are representative frames. The “3D” in the panels refer to 3-D reconstructions of the areas indicated by the white boxes, calculated from the *z*-stacks. Here, the reconstructions are observed from the top. To view a complete rotation on the *x*-axis of the 3D reconstructions, see Videos S4 and S5. (B) Side-view of the *z*-stack in (A), cut along the white line, showing a Gα_s_-positive tubule ending in a vesicle positive for both Gα_s_ and TSH-Alexa594. Throughout the figure, yellow in the merged images is indicative of colocalization. Images in (A–C) are representative of 25–30 cells per condition analyzed in at least three independent experiments.

To identify the structures that were positive for Gα_s_, we performed a series of colocalization experiments with markers of intracellular compartments. First, we used a fluorescent transferrin conjugate to label early endosomes and the recycling endosomal compartment. This is a frequently used method that takes advantage of the endocytosis and recycling of transferrin together with its receptor [40]. To this end, primary mouse thyroid cells were stimulated with fluorescent transferrin for various periods of time (2–60 min), followed by fixation and immunofluorescent staining of Gα_s_. Early time points (2–5 min) were used to visualize early endosomes, whereas later time points were employed to identify recycling endosomes. Gα_s_ and transferrin were not found simultaneously on early endosomes (unpublished data). However, at later time points (20–60 min) transferrin appeared to be contained in vesicles, presumably a recycling compartment, associated with the perinuclear tubulovesicular structure positive for Gα_s_ (Figure 10A). Of note, some of these vesicles were also positive for Gα_s_. In addition, primary mouse thyroid cells were simultaneously treated with fluorescent transferrin and TSH to evaluate whether they followed similar or distinct endocytic pathways (Figure 10B and unpublished data). A partial colocalization between internalized TSH and transferrin was observed at all time points considered (2–60 min)—notice that in Figure 10B, several TSH-positive vesicles contain also transferrin—suggesting that they followed to some extent the same trafficking pathway. An antibody against Rab7 was used to label late endosomes. No colocalization was observed between Gα_s_ and Rab7 (Figure 10C). Finally, an antibody against a Golgi-resident protein, Golgi 58K, was used to mark the Golgi compartment [41]. A high degree of colocalization was present between Gα_s_ and Golgi 58K (Figure 10D), suggesting that a relevant fraction of Gα_s_ was located on membranes of the Golgi complex.

Next, we treated primary thyroid cells with TSH-Alexa594, as a marker of TSH receptor, and looked at its colocalization with Gα_s_ and adenylyl cyclases. Interestingly, internalized TSH and Gα_s_ were frequently found in close association, with TSH being present on vesicles adjacent to the Gα_s_-positive tubulovesicular structure (Figure 11A and 11B and Video S4). As observed in the case of transferrin, some of these vesicles were also positive for Gα_s_. TSH and adenylyl cyclase III were found to colocalize occasionally on the plasma membrane and more frequently on intracellular vesicles or tubulovesicular structures (Figure 11C and Video S5). Little colocalization was observed between internalized TSH and adenylyl cyclase V/VI (unpublished data).

**Figure 12:**
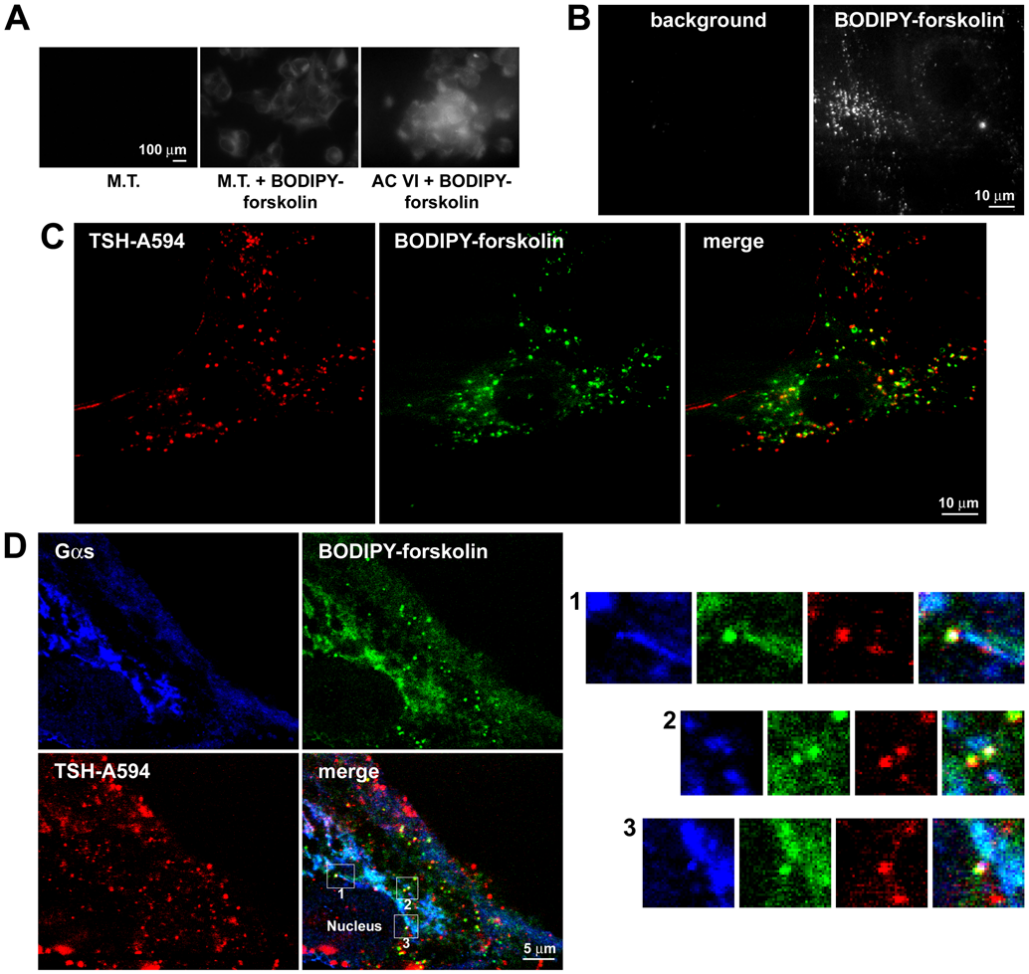
BODIPY-forskolin labeling of adenylyl cyclases. (A) Test experiment in HEK293 cells. HEK293 cells were either transfected with canine adenylyl cyclase VI cDNA (AC VI) or mock transfected (M.T.). Forty-eight hours after the transfection, they were stained with BODIPY-forskolin and directly visualized with a fluorescent microscope. Note the higher staining in cells overexpressing adenylyl cyclase VI. (B) BODIPY-forskolin labeling of primary thyroid cells. Mouse primary thyroid cells were stained with BODIPY-forskolin and visualized with a TIRF microscope set to have a high penetration depth. (C) Live-cell imaging of adenylyl cyclases and internalized TSH in primary thyroid cells. Primary mouse thyroid cells were stimulated with 3 µg/ml TSH-Alexa594 for 20 min, followed by 10 min staining with BODIPY-forskolin. TSH-Alexa594 and BODIPY-forskolin were visualized with a TIRF microscope as above. A frequent colocalization between TSH-Alexa594 and BODIPY-forskolin on intracellular vesicles and small tubulovesicular structures was observed. (D) Triple staining for adenylyl cyclases, Gα_s_, and TSH. Mouse primary thyroid cells were stimulated with 3 µg/ml TSH-Alexa594 for 20 min, fixed, and then processed for Gα_s_ immunofluorescence. Immediately before imaging, the coverslips were mounted in an experimental chamber, stained with BODIPY-forskolin, and directly visualized with a confocal microscope. White is indicative of triple colocalization. Images in (A) are representative of three independent experiments. Images in (B–D) are representative of more than 20 cells per condition analyzed in at least three independent experiments.

To further investigate the subcellular localization of adenylyl cyclases, we labeled them with BODIPY-forskolin, a fluorescent forskolin analog [42]. The specificity of BODIPY-forskolin staining was initially evaluated in HEK293 cells transfected with adenylyl cyclase VI cDNA, which showed a stronger labeling compared to control mock-transfected cells (Figure 12A). Then, we utilized BODIPY-forskolin to stain primary thyroid cells that were visualized with a TIRF microscope as described above. BODIPY-forskolin staining was largely present on intracellular vesicles and tubulovesicular structures (Figure 12B). Utilizing this approach, we could also visualize simultaneously the internalized TSH-Alexa594 and BODIPY-forskolin in cells that were stimulated with the fluorescent ligand. A consistent colocalization was observed at the level of several intracellular vesicles and tubulovesicular structures, in agreement with the previous immunofluorescence data (Figure 12C). The dynamic nature of these intracellular structures can be appreciated in Video S6. Finally, we utilized the fluorescent forskolin analog to perform triple stainings in which we attempted to simultaneously visualize the internalized TSH-Alexa594, Gα_s_ (by immunofluorescence), and adenylyl cyclases. The resulting images were indicative of the three components being simultaneously present on vesicles that were often adjacent to the perinuclear tubulovesicular structure positive for Gα_s_ (Figure 12D). Of note, the latter structure was also labeled by BODIPY-forskolin (Figure 12D).

Finally, we utilized immunogold electron microscopy to evaluate the subcellular localization of the internalized TSH, Gα_s_, and adenylyl cyclase III at the ultrastructural level. None of the available antibodies against Gα_s_ worked in immunogold stainings. To visualize the internalized TSH, we labeled it with Alexafluor488, which was recognized by a specific antibody. Similarly to TSH-Alexa594, TSH-Alexa488 had a conserved biological activity (unpublished data). Consistent with the previous immunofluorescence results, both TSH-Alexa488 and adenylyl cyclase III were found to be present in endosomes (Figure S7).

### Effect of Receptor Internalization on cAMP Signaling

**Figure 13:**
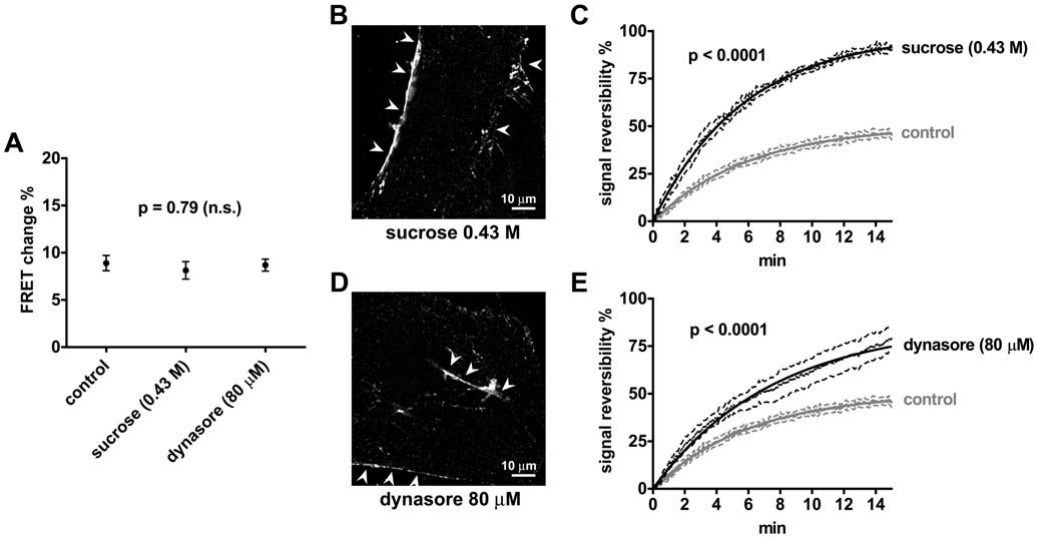
Effect of endocytosis inhibition on cAMP signaling. Cells were prestimulated with 0.43 M sucrose for 10 min, 80 µM dynasore for 20 min, or normal medium as control. (A) Comparison of FRET changes induced by stimulating thyroid follicles obtained from CAG-Epac1-camps mice with TSH (30 U/l for 2 min, as in Figure 5C) in the presence or absence (control) of endocytosis inhibitors (n = 6–8 per each condition). Error bars indicate SEM. (B) Confocal image of a primary mouse thyroid cell stimulated with TSH-Alexa594 (3 µg/ml for 20 min) in the presence of 0.43 M sucrose. Note the binding of TSH-Alexa594 to the plasma membrane (arrowheads) and the almost complete inhibition of TSH-Alexa594 internalization (no intracellular vesicles). For comparison, see Figure 8 (20 min). (C) Comparison of cAMP signal reversibility after TSH stimulation (30 U/l for 2 min) in the presence or absence (control) of 0.43 M sucrose (n = 6, each). (D) Confocal image of a primary mouse thyroid cell stimulated with TSH-Alexa594 (3 µg/ml for 20 min) in the presence of 80 µM dynasore, showing consistent inhibition of TSH-Alexa594 internalization. Arrowheads, TSH-Alexa594 bound to the plasma membrane. (E) Comparison of cAMP signal reversibility after TSH stimulation (30 U/l for 2 min) in the presence or absence (control) of 80 µM dynasore (n = 6, control; n = 8, dynasore). Signal reversibility in (C) and (E) is calculated as in Figure 5F. Fits were compared with F test, having a null hypothesis that *Y*_max_ values were the same for all datasets. Images in (B) and (D) are representative of more than 20 cells per condition analyzed in three independent experiments.

Although our data were so far compatible with TSH receptor signaling from intracellular sites, direct functional evidence was still missing. We therefore reasoned that if our hypothesis was correct, treatments capable of inhibiting TSH receptor endocytosis should hamper TSH receptor sequestration and, as a result, increase the reversibility of cAMP signals after TSH washout. We first tried several treatments that have been reported to inhibit GPCR internalization, such as concanavalin A, phenylarsine oxide and hypertonic sucrose [7]. Concanavalin A and phenylarsine oxide were found to partially inhibit forskolin-dependent cAMP production and, therefore, were not further evaluated (unpublished data). By contrast, pretreatment with 0.43 M sucrose for 10 min did not hamper TSH-dependent cAMP accumulation (Figure 13A), whereas it almost completely inhibited TSH-Alexa594 internalization (Figure 13B, Video S7 and Video S8). Importantly, pretreatment of thyroid follicles with hypertonic sucrose was associated with a complete reversibility of the cAMP signal produced by TSH stimulation for 2 min (Figure 13C). The mechanism of action of hypertonic sucrose is not completely understood, though this treatment is known to alter the polymerization of clathrin, thus hampering the formation of coated pits at a very initial phase [7]. Another frequently used method to inhibit GPCR internalization is to interfere with dynamin function. Dynamin is in fact required for later phases of clathrin-dependent endocytosis, such as pinching and release of completed coated pits from the plasma membrane. Recently, Macia et al. developed a membrane permeable dynamin inhibitor, dynasore [43], which could be applied directly to intact thyroid follicles. We therefore repeated the same experiments in the presence of dynasore, which had a consistent inhibitory effect on TSH internalization (Figure 13D and Video S9). Analogously to what was observed with hypertonic sucrose, pretreatment for 20 min with 80 µM dynasore, a concentration that produced a maximal effect on TSH internalization, resulted in a major increase of cAMP signal reversibility after TSH washout (Figure 13E). Taken together, these results strongly suggested that TSH receptor internalization was responsible for the observed irreversibility of cAMP signaling.

### Effect of pH on TSH Receptor-cAMP Signaling

A possible concern when considering the occurrence of TSH receptor signaling on endocytic membranes is the effect of acidic pH on TSH–TSH receptor interactions. In fact, the low pH of early (pH 5.9–6.2) and late endosomes (pH.5.0–6.0) [44] is known to promote the dissociation of several receptor–ligand complexes. To evaluate the effect of acidic pH on TSH receptor signaling, we incubated primary thyroid follicles at different pH levels and evaluated the cAMP response to TSH stimulation by FRET microscopy. In agreement with previous observations [45], our results suggested that efficient TSH binding and activation of the TSH receptor was still possible at pH 5.0 (Figure S8).

### Cell Fractionation Experiments

To further verify the hypothesis that the TSH receptor may continue to signal to Gα_s_/adenylyl cyclase after internalization, we conducted a series of experiments based on cell fractionation. Since in initial tests we found that these studies required a higher amount of starting material than could be obtained from primary thyroid cells, these experiments were performed on the FRTL5 thyroid cell line. FRTL5 cells are a widely used model of well-differentiated thyroid cells, which, among other features of normal thyrocytes, express thyroid-specific genes, conserve a functional TSH receptor-cAMP signaling pathway and retain the capability to synthesize thyroid hormones [28],[46].

**Figure 14:**
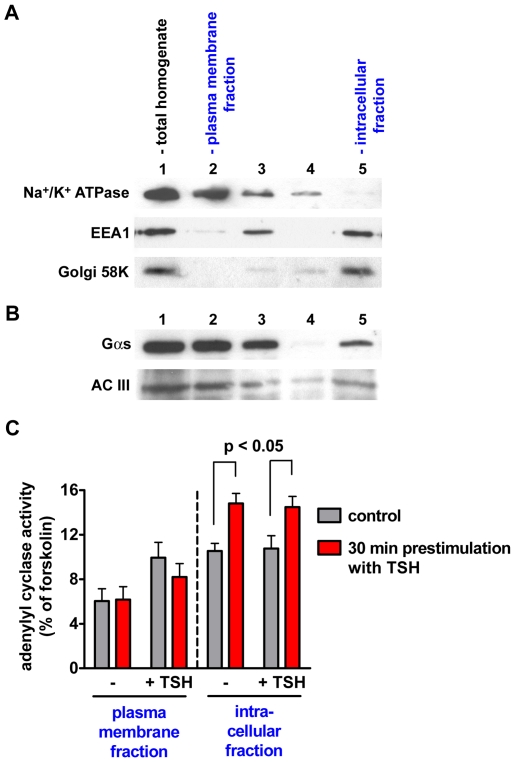
Cell fractionation experiments. The plasma membrane and the intracellular fractions of FRTL5 cells were obtained by separation with concanavalin A-coated magnetic beads. (A) Western blot analysis of subcellular markers in the obtained fractions. The following markers were used: Na^+^/K^+^ATPase for the plasma membrane, the early endosome antigen 1 (EEA1) for early endosomes, and Golgi 58K for the Golgi complex. 1, total homogenate. 2, first eluate from the magnetic beads, corresponding to the plasma membrane fraction. 3, postnuclear supernatant. 4, second eluate from the magnetic beads. 5, intracellular fraction. (B) Western blot for Gα_s_ and adenylyl cyclase III (AC III) in the same fractions as in (A). (C) Effect of TSH stimulation on adenylyl cyclase activity in the subcellular fractions. FRTL5 cells were starved for 24 h in medium without TSH and either stimulated with 30 U/l TSH for 30 min or mock stimulated (control), followed by cell fractionation with concanavalin A-coated magnetic beads. The adenylyl cyclase activity in the plasma membrane and intracellular fractions was then determined in the absence of stimuli (−) or in the presence of either 30 U/l TSH (+TSH) or 10 µM forskolin. The results were normalized for the maximal adenylyl cyclase activity measured in the presence of forskolin. Shown are the data from three independent experiments. Error bars indicate SEM.

First, we established a method to separate the intracellular content from the plasma membrane. In order to allow a better characterization of the supposed intracellular TSH receptor signaling compartment, we looked for a method that was fast, as gentle as possible, and produced with high yield an intracellular fraction with the lowest possible contamination from the plasma membrane. The best results were obtained utilizing a protocol based on the separation of the plasma membrane with magnetic beads coated with concanavalin A, which binds selectively to the glycoproteins present on the cell surface [47]. Figure 14A shows the results of a typical fractionation experiment. Please notice the virtual absence of the Na^+^/K^+^ ATPase, used as a plasma membrane marker, in the intracellular fraction after the second round of purification (lane 5).

Next, we evaluated by Western blot analysis the presence of Gα_s_ and adenlyl cyclase III in the different subcellular fractions (Figure 14B). As expected, bands corresponding to Gα_s_ and adenylyl cyclase III were present in the total homogenate as well as in the plasma membrane fraction. In addition, the same bands were also present in the intracellular fraction, thus confirming the evidence based on the results of the immunofluorescence experiments that both Gα_s_ and adenylyl cyclase III were present in significant amounts also in the intracellular compartment.

Once the fractionation method was established, we attempted to directly measure the activity of adenylyl cyclase in the plasma membrane and in the intracellular fraction of cells that were previously stimulated with TSH. The prediction was that if the TSH receptor continued to signal to Gα_s_/adenylyl cyclase after internalization, this should result in an increase of the adenylyl cyclase activity in the intracellular fraction. This appeared to be indeed the case, as we found that pretreatment of FRTL5 cells for 30 min with TSH was associated with an increased adenylyl cyclase activity in the intracellular fraction (Figure 14C). By contrast, pretreatment with TSH was not associated with any modifications of the adenylyl cyclase activity in the plasma membrane fraction. This is not surprising, as TSH is expected to dissociate from the TSH receptors present on the plasma membrane during the time required for the cell fractionation. Nevertheless, a certain amount of functional receptors was left on the plasma membrane, as adenylyl cyclase activity in this fraction could be increased by adding TSH in the reaction mixture (Figure 14C).

Altogether, these results further supported the hypothesis that the TSH receptor continued to signal to Gα_s_/adenylyl cyclase after internalization.

### Simulations

**Figure 15:**
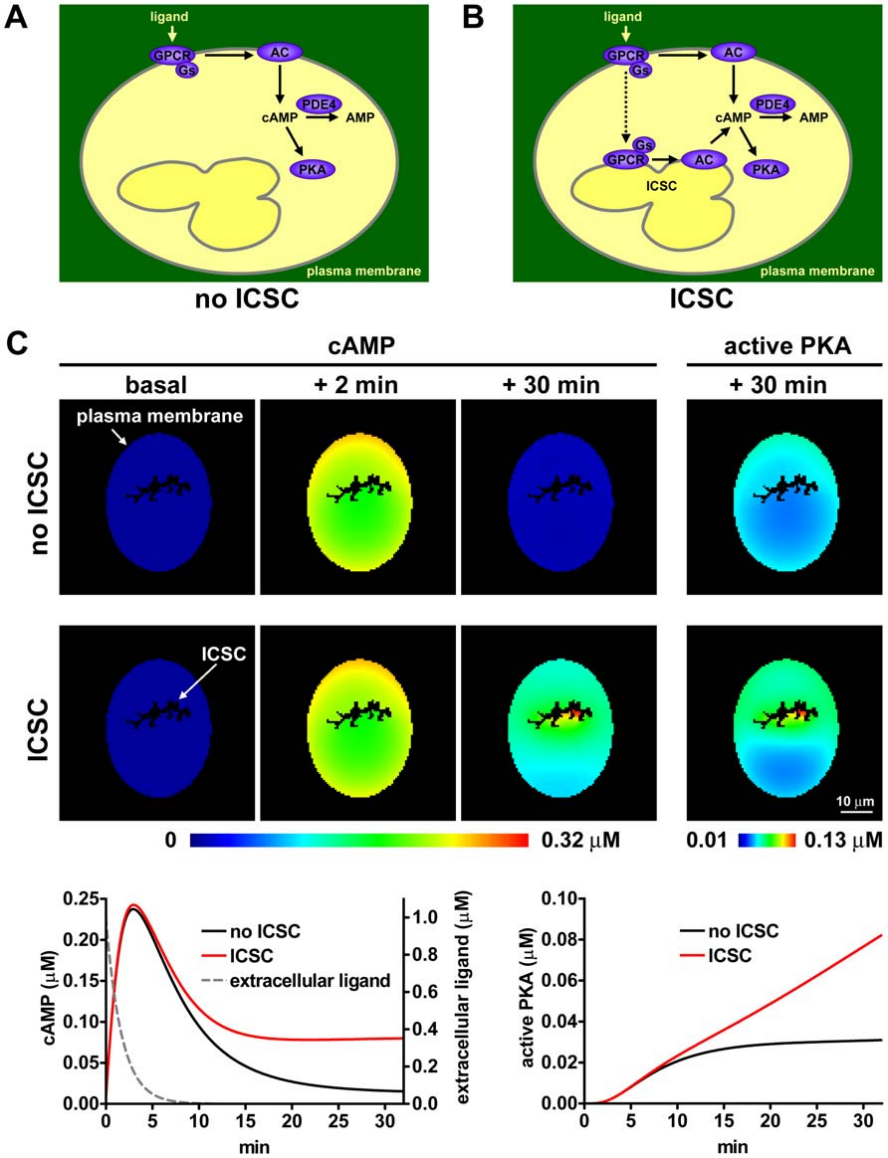
Mathematical model of the GPCR-cAMP signaling pathway. A model of spatial partial differential equations was generated to simulate the temporal and spatial dynamics of GPCR signaling. (A) Schematic illustration of the basic model. The receptor, G-proteins, and adenylyl cyclase are placed on the plasma membrane, whereas ATP, cAMP, and PDE4 are freely diffusing in the cytosol. PKA is cytosolic, but nondiffusing. (B) Model with the addition of an intracellular signaling compartment (ICSC). To simulate GPCR-cAMP signaling from an ICSC, we placed G-proteins and adenylyl cyclase also on an intracellular membrane and simulated the internalization of both GPCR and ligand to this compartment. (C) Results of simulations. A cell was transiently stimulated by application and removal of the ligand from the extracellular compartment. In a first simulation in which signaling from the ICSC was not implemented (no ICSC), the cAMP response was completely reversible. On the contrary, inclusion of the ICSC in the model lead to sustained cAMP production. Also note the different levels and spatial patterns of PKA activation predicted in the presence or absence of the ICSC.

To better understand the possible consequences of intracellular GPCR signaling, we generated a mathematical model of the GPCR-cAMP pathway. Our model is based on the recent work done by Neves et al. on β_2_-adrenergic receptor signaling [21]. In this model, the receptor, G-proteins and adenylyl cyclase are placed on the plasma membrane, whereas ATP, cAMP, PDE4, and PKA are cytosolic (Figure 15A). In addition, to mimic an intracellular signaling compartment (ICSC), we placed G-proteins and adenylyl cyclase also on an intracellular membrane and simulated the internalization of both GPCR and ligand to this compartment (Figure 15B). Simulations were performed with the Virtual Cell software [48]–[50]. Initial non-spatially resolved simulations, aimed at validating the model and assessing the effects of different geometries and parameters as well as of receptor recycling, are described in Text S1 and Figures S9, S10, S11. Subsequently, a system of partial differential equations was used to obtain a spatiotemporal description of the GPCR-cAMP pathway. The results of the simulations performed with this model were in good agreement with our experimental data. In Figure 15C are reported the results of a spatial simulation in which a cell was given a transient stimulus, by mimicking a perfusion system that provided and subsequently removed the ligand from the extracellular space. Notice that in the absence of the ICSC, the cAMP response is fully reversible. On the contrary, in the presence of the ICSC, the signal is no longer reversible, in agreement with the results of the FRET experiments. Interestingly, the different types of cAMP gradients generated in the presence or absence of the ICSC are also reflected by different degrees and spatial patterns of PKA activation. Thus, our mathematical model predicted that GPCR signaling to cAMP from an ICSC should have important consequences also on the spatiotemporal dynamics of downstream signaling events.

### Functional Consequences of cAMP Signaling from Internalized Receptors

**Figure 16:**
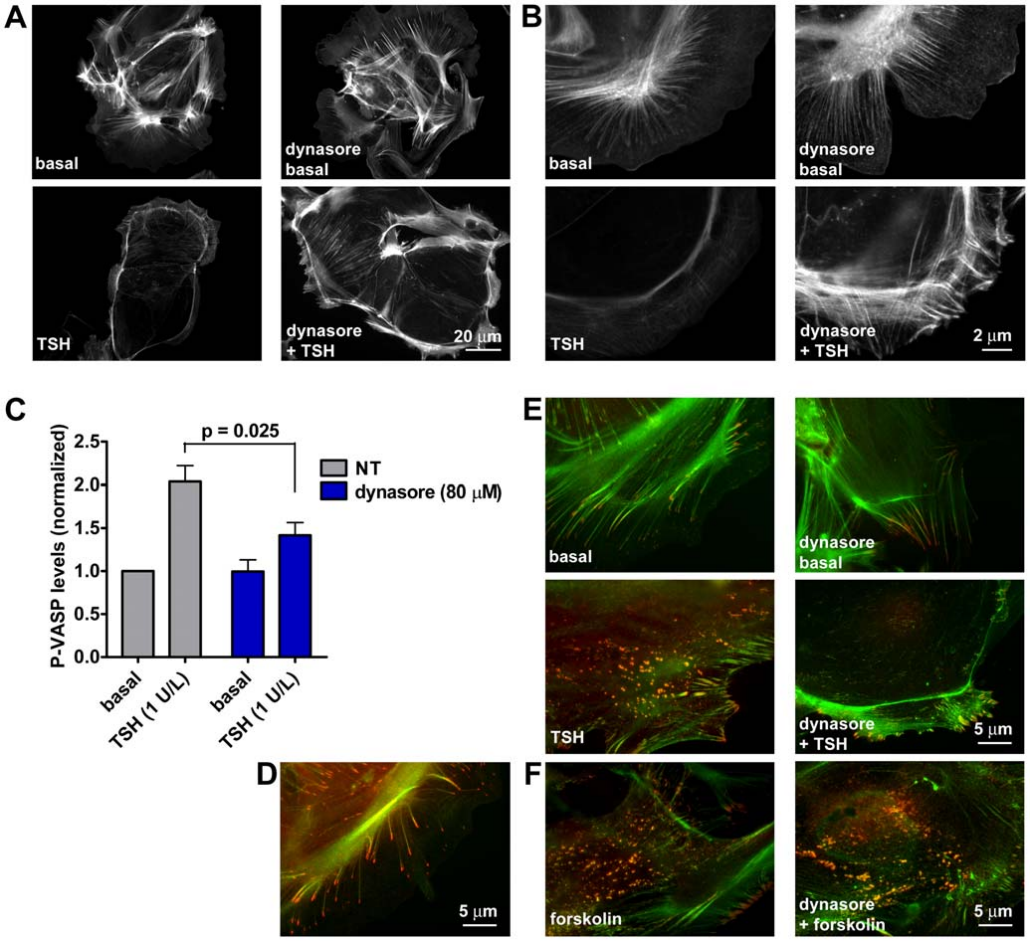
Effect of endocytosis inhibition on downstream signaling. (A and B) Actin depolymerization in response to TSH. Mouse primary thyroid cells were preincubated with normal medium or medium plus 80 µM dynasore for 20 min and stimulated with 30 U/l TSH for an additional 20 min in the presence or absence of dynasore as indicated. Cells were then fixed, and actin was stained with fluorescent phalloidin. Note that dynasore largely prevented the depolymerization of actin in response to TSH. (B) High-magnification images of actin rearrangement in lamellipodia, where the effect of dynasore was more pronounced. (C) VASP phosphorylation. Primary mouse thyroid cells were preincubated with normal medium or medium plus 80 µM dynasore for 20 min. Cells were then stimulated with 1 U/l TSH for 30 min, in the presence or absence of dynasore as indicated. Levels of P-VASP (Ser 157) and total VASP were evaluated by Western blot analysis. Shown are the mean P-VASP levels of three independent experiments. Error bars indicate SEM. (D) Subcellular localization of VASP. Mouse primary thyroid cells were labeled by immunofluorescence with an antibody against total VASP (red) together with fluorescent phalloidin to stain actin (green). Shown is a merged fluorescent image. VASP is typically located at the ends of actin filaments. (E) Pattern of VASP phosphorylation in response to TSH. Mouse primary thyroid cells were preincubated and stimulated with TSH in the presence or absence of dynasore as explained above. Cells were then labeled by immunofluorescence with an antibody against VASP phosphorylated at Ser 157 (red) together with fluorescent phalloidin to stain actin (green). Note the appearance of spots containing phosphorylated VASP and actin in the central cellular compartment only in the absence of dynasore. (F) Actin depolymerization and pattern of VASP phosphorylation in response to forskolin. Cells were treated as in (E), with the exception that instead of TSH, they were stimulated with 10 µM forskolin. Note a similar degree of actin depolymerization and a similar pattern of VASP phosphorylation both in the presence and in the absence of dynasore. Images in (A and B) and (D–F) are representative of at least three independent experiments.

Based on the results of the mathematical simulations, we investigated whether the inhibition of TSH receptor internalization had some effects on the signaling events downstream of cAMP production. One of the earliest effects of TSH in thyroid cells is the reorganization of the actin cytoskeleton. This is a well-known phenomenon, mainly consisting in the depolymerization of stress fibers, that is implicated in the reuptake of thyroglobulin and in the induction of thyroid-specific genes [51]–[53]. Therefore, we evaluated whether blocking receptor internalization might affect the depolymerization of actin in response to TSH (Figure 16A and 16B). Primary mouse thyroid cells were stimulated with TSH in the presence or absence of endocytosis inhibitors, as described above, then fixed and stained for actin with fluorescent phalloidin. Hypertonic sucrose itself was found to induce a modification of actin architecture, and was not further evaluated (unpublished data). Instead, dynasore treatment did not cause any appreciable modifications of cellular shape or actin polymerization (Figure 16A). As expected, TSH alone caused a generalized depolymerization of actin, which was observed both in the central cellular compartment (Figure 16A) and in lamellipodia (Figure 16B). By contrast, in the presence of dynasore, TSH induced only a partial depolymerization in the central cellular compartment (Figure 16A) and failed to induce depolymerization in lamellipodia, which showed an even thicker actin mesh compared to that of control cells (Figure 16B).

The PKA-substrate vasodilator-stimulated phosphoprotein (VASP) is a key effector of cAMP in the reorganization of actin cytoskeleton [54],[55]. VASP is concentrated at actin hot spots (lamellipodia, filopodia, cell–substrate, and cell–cell contacts) where it regulates the polymerization and branching of actin filaments. Importantly, its function is regulated by PKA via phosphorylation at Ser 157 [54],[55]. We therefore investigated whether VASP was phosphorylated in response to TSH stimulation in thyroid cells and the possible consequences of TSH receptor internalization on this pathway. VASP phosphorylation was evaluated by Western blot analysis, both by monitoring the intensity of the slower migrating band detected by a VASP antibody and by utilizing an antibody specific for VASP phosphorylated at Ser 157. In the absence of endocytosis inhibitors, TSH caused a 2-fold induction of VASP phosphorylation. However, pretreatment with endocytosis inhibitors caused a substantial reduction of VASP phosphorylation in response to TSH (Figures 16C and S12).

To confirm the possible involvement of VASP in the control of actin polymerization in thyroid cells, we examined its subcellular localization by immunofluorescence. As observed in other cells in which VASP regulates actin polymerization, VASP was concentrated at the ends of actin filaments (Figure 16D).

Finally, we evaluated whether blocking TSH receptor internalization also altered the spatial pattern of VASP phosphorylation by PKA. To this end, we stimulated thyroid cells with TSH alone or in the presence of dynasore, and visualized VASP phosphorylated at Ser 157 by immunofluorescence (Figure 16E). In the absence of dynasore, TSH caused a robust increase of VASP phosphorylation throughout the cell. In particular, phosphorylated VASP colocalized with spots of depolymerized actin in the central cellular compartment. By contrast, only a minor induction of VASP phosphorylation and no spots of phosphorylated VASP in the central cellular compartment were observed in the presence of dynasore (Figure 16E). As a control, the generalized depolymerization of actin and the pattern of VASP phosphorylation induced by forskolin were not modified by dynasore (Figure 16F).

Taken together, these results suggest that TSH receptor internalization not only modifies the temporal dynamics of cAMP signaling by leading to persistent cAMP production, but may also affect the intensity and the spatial patterning of downstream signals.

## Discussion

The conventional model of GPCR signaling is based on the central concept that signaling to second messengers such as cAMP is taking place only at the cell plasma membrane [1]. The role assigned to receptor internalization is essentially to reduce the number of GPCRs present on the cell surface, thus contributing to signal desensitization, or to bring the receptors to an intracellular site for dephosphorylation and resensitization [1],[2],[56]. Whatever the fate of the internalized GPCRs, they are thought to stop signaling to second messengers once inside the cell. Here, we provide evidence that a GPCR continues to stimulate cAMP production after internalization. cAMP signaling by internalized receptors appears to be different from that occurring at the plasma membrane, as it is of a sustained nature and leads to a different pattern of downstream signals.

Although the biochemical steps involved in GPCR signaling are known in detail, their location in space and time in living cells is poorly understood. This represents a major drawback, as there is emerging evidence that signaling cascades are highly organized in space and time, and intrinsically dynamic [20]. On such a basis, there is an urgent need to develop new tools and techniques to monitor signaling events with submicrometer resolution and fast temporal dynamics. Recently, the application of FRET to color variants of *Aequorea victoria* GFP has allowed the development of a toolbox of genetically encoded sensors to observe intracellular signaling events in real time. Several sensors have been described that exhibit FRET changes on exposure to cAMP [57]. A sensor based on the dissociation of the PKA subunits was first described by Adams et al. (using rhodamine and fluorescein as fluorophores) [58] and later modified by Zaccolo and colleagues to be genetically encoded by substituting rhodamine and fluorescein with GFP variants [12],[13]. This sensor has led to major insights into the biology of cAMP [13],[25],[59]–[61]. We have recently described another type of sensors, including Epac1-camps, that are based on a single cAMP-binding domain derived from cAMP-binding proteins [14],[26]. These sensors, being based on a cAMP-binding domain alone and not on the entire protein, are devoid of any signaling activity. Therefore, they are extremely well tolerated and are expected not to alter the functions of the cell [57]. This is probably why the CAG-Epac1-camps mice are viable and healthy, whereas attempts to create genetically modified organisms with ubiquitous expression of PKA-based sensors have so far been unsuccessful [62],[63].

The newly generated cAMP reporter mice were used to monitor GPCR signaling in living cells. A major strength of our study was the use of a highly physiological system, i.e., 3-D thyroid follicles. Thyroid follicles represent a unique model to study the spatiotemporal dynamics of cAMP signaling because they maintain the supracellular organization, size, and polarization possessed by thyroid cells in vivo, and constitute a rare example of cells that are under the strict control of a GPCR (the TSH receptor) and of cAMP for virtually all their functions (e.g., thyroid hormone production, cell proliferation) [27],[28]. The conservation of the original cellular architecture of thyroid tissue is reflected by the high degree of spatial organization of the TSH receptor-cAMP signaling cascade: the TSH receptor is expressed on the basolateral membrane; upon binding of TSH, cAMP produced at the basolateral membrane diffuses through the cytosol to activate cytosolic PKA I and PKA II, mainly located in the Golgi complex; PKAs in turn phosphorylate targets localized in different cellular compartments (e.g., cytosol, nucleus, as well as apical and basolateral membranes) [27],[28],[64]–[66]. The maintenance of the original cellular size and shape is of fundamental importance, as modifications of these parameters are expected to alter the properties of signaling cascades, including the shape of the gradients of cAMP and other soluble messengers [20]–[22]. Another advantage of thyroid follicles is that they already express all the required signaling machinery at endogenous levels. Here, we describe a method to directly visualize cAMP signaling in thyroid follicles, thus allowing, for the first time to our knowledge, a precise monitoring of the kinetics of a GPCR-cAMP signaling cascade, at endogenous levels of expression and in its native multicellular functional unit. We believe that this represents a major step towards depicting signaling pathways in their natural context.

By monitoring cAMP signaling and receptor internalization, we obtained a series of unexpected findings. First, the TSH receptor internalizes rapidly and consistently into a pre-Golgi compartment in close association with Gα_s_ and adenylyl cyclase III. Second, the robust internalization of the TSH receptor is not associated with any appreciable desensitization of the cAMP signal. Third, prolonged TSH receptor stimulation leads to a sustained production of cAMP; by contrast, short stimuli are completely reversible. Fourth, blocking receptor internalization can prevent the irreversibility of cAMP signals. Fifth, TSH receptor internalization is required to ensure a normal pattern of actin rearrangement and VASP phosphorylation downstream of cAMP. On the basis of these findings, we suggest that the TSH receptor continues to signal to adenylyl cyclase after internalization, and that the location of TSH receptor-cAMP signaling affects the spatial patterns of the downstream signals.

The consequences of GPCR signaling from inside the cell appear to be multiple, as anticipated by Miaczynska et al. [4]. First, sustained cAMP production from internalized receptors may provide a memory mechanism, allowing thyroid cells to maintain constant thyroid hormone production in the presence of fluctuations in plasma TSH concentration. Indeed, TSH release from the pituitary follows a circadian rhythm, with a zenith in the first hours of the morning, but those fluctuations are not associated with a change in the production of thyroid hormones [67]. Additionally, intracellular membranes may provide specialized platforms for signal compartmentalization. This is the case of MAPK activation by GPCRs, which appears to happen selectively on endosomes [7], and of tyrosine kinase receptors that activate a distinct subset of substrates once inside the cell [3]–[6]. In the case of cAMP signaling, internalized receptors appear to be more efficiently coupled to PKA, as suggested by the effect of endocytosis inhibitors on VASP phosphorylation. This could be explained if the receptors need to be brought close to an intracellular pool of PKA for efficient kinase activation and/or VASP phosphorylation.

The existence of spatial microdomains of cAMP signaling inside the cell has been debated for years. Unlike Ca^++^, whose apparent diffusion is limited by the high buffering capacity of cytosolic proteins [68],[69], measurement of cAMP diffusion gave values in the range of 270–780 µm^2^/s, i.e., as fast as would be expected in a simple electrolyte solution [70],[71]. For this reason, cAMP has been traditionally considered a far-reaching messenger, capable of crossing the whole cell to convey the information generated at the plasma membrane. Although this might be the case in certain circumstances, as for example, in the case of long-term facilitation in *Aplysia* sensory neurons [72], it is difficult to reconcile the free diffusion of cAMP with the high spatial organization of the cAMP signaling cascade (adenylyl cyclases, PKAs, AKAPs, PDEs, etc.) [11],[57],[59],[72]–[78]. This paradox has been partially resolved by recent reports of restricted cAMP diffusion. A striated pattern of cAMP signaling has been observed in cardiomyocytes with a genetically encoded PKA-based sensor [13]. In addition, a study by our group has revealed that in these cells, β_1_-adrenergic receptors produce generalized cAMP responses, whereas β_2_-adrenergic receptors generate locally confined signals [26]. Furthermore, the existence of cAMP microdomains is supported by a body of indirect evidence [15],[59],[75],[79]–[86], and the formation of cAMP gradients is predicted on the basis of the spatial segregation of adenylyl cyclases on the membrane and PDEs in the cytosol [20],[22],[87]. Recent calculations suggest that such gradients may have a length of 2.5–4 µm [22]. If this is indeed the case, signals originating near the plasma membrane could hardly reach deep inside the cell. In this perspective, GPCR-cAMP signaling from intracellular sites might provide a new basis to explain the activation of distant targets and the specific effects observed with different types of activation.

In an attempt to identify the intracellular compartment(s) where sustained TSH receptor-cAMP signaling was occurring, we analyzed the subcellular localization of the internalized TSH, Gα_s_, and adenylyl cyclases by a combination of experimental approaches. In agreement with previous observations [31], our results indicate that TSH and its receptor are internalized rapidly in endosomes. Later on, at least part of the internalized TSH is found together with transferrin in perinuclear vesicles that probably represent a recycling endosomal compartment. Interestingly, some of these vesicles appear to contain also Gα_s_ and adenylyl cyclases. Although we cannot exclude the possibility that subdetectable levels of Gα_s_ may be present together with adenylyl cyclases in early endosomes and that TSH receptor-cAMP signaling might be taking place also in this compartment, those perinuclear vesicles that simultaneously contain TSH, Gα_s_, and adenylyl cyclases are the most likely candidates to be the intracellular sites of TSH receptor-cAMP signaling. An interesting aspect that emerges from our experiments and that should be taken into consideration is the highly dynamic nature of the intracellular structures where the internalized TSH, adenylyl cyclases, and Gα_s_ are present. These compartments appear in fact highly complex, sometimes having specialized subdomains that prevalently contain one component and rapidly exchange their contents by fusing to each other or through budding of new vesicles. Additional studies, probably requiring the generation of better antibodies and new tools to simultaneously monitor the localization of the receptor, Gα_s_ and adenylyl cyclases in real time, are needed to further investigate the microscopic anatomy and the dynamic nature of the intracellular cAMP signaling compartment(s).

In summary, our data show that GPCR signaling in a physiological setting may be more complex than current knowledge, based mostly on studies with transfected cells, suggests. In particular, the subcellular localization of GPCRs, either on the plasma membrane or intracellular, appears to be an important parameter affecting the duration and the spatial pattern of downstream signals. These findings may lead to reconsidering the current model of GPCR signaling and suggest new and intriguing scenarios on the function of GPCRs inside the cell.

## Materials and Methods

### Ethics Statement

All animal work was done according to the regulations and with the permission of the government of Lower Franconia.

### Materials and Reagents

Cell culture media and reagents were from Pan Biotech. Glass-bottom Petri dishes were from World Precision Instruments. Collagen and dispase were from Roche Diagnostics. Bovine TSH (bTSH), dynasore, the mouse monoclonal antibody against Golgi 58K protein, the cAMP enzyme immunoassay kit (CA200), and alumina WN-6 columns were from Sigma-Aldrich; 7-deacetyl-7-[O-(N-methylpiperazino)-γ-butyryl)]-forskolin (DMPB-forskolin) was from Merck. Rabbit polyclonal antibodies against Gα_s_, adenylyl cyclase III, and adenylyl cyclase V/VI were from Santa Cruz Biotechnology. Antibodies against Rab7 (mouse monoclonal), Na^+^/K^+^ ATPase (mouse monoclonal), and EEA1 (rabbit polyclonal) were from Abcam. Antibodies against VASP (rabbit monoclonal) and P-VASP (Ser 157, rabbit polyclonal) were from Cell Signaling Technology. Cy2-conjugated anti-rabbit and anti-mouse polyclonal antibodies were from Jackson ImmunoResearch. Collagenase I and II, Alexafluor488 and Alexafluor594 succinimidyl esters, the Alexafluor594-conjugated goat anti-rabbit polyclonal antibody, Alexafluor488-conjugated transferrin, Alexafluor488-conjugated phalloidin, the rabbit anti-Alexafluor488 antibody, and Dynabeads Biotin Binder magnetic beads were from Invitrogen. The goat anti-rabbit and anti-mouse antibodies conjugated with horseradish peroxidase were from Amersham Pharmacia Biotech and Millipore. The ECL detection kit was from Amersham Pharmacia Biotech. Effectene transfection reagent was from Qiagen. Biotinylated concanavalin A was from Vector Laboratories. [α-^32^P]ATP was from PerkinElmer Life Sciences. All other reagents were from Sigma-Aldrich.

### Generation and Characterization of Transgenic Mice

To generate transgenic cAMP-sensor mice, we followed the strategy used for GFP mice [23]. FVB one-cell embryos were injected by standard procedures with a genetic construct in which the sensor sequence was inserted between the CAG promoter and the rabbit β-globin polyadenylation signal (See Figure 1A). The original pCAGGS expression vector has been described and provided by J. Miyazaki [88]. The screening of pups for transgene insertion was performed by PCR analysis as previously described [26]. Three transgenic lines were obtained that showed different levels of fluorescence. The line with the highest expression was used for further experiments. Mice and freshly isolated organs were imaged with a Leica macroFluo (Z6APO-A) microscope, using the YFP emission filter.

### Cell Culture

Mouse thyroid follicles were isolated according to a previously published protocol [30], with minor modifications. Thyroid lobes were dissected from 1–2 mo-old mice. The lobes were collected in a 1.5-ml microcentrifuge tube, containing 1 ml of digestion medium, which consisted of 100 U/ml collagenase I, 100 U/ml collagenase II, and 1 U/ml dispase, dissolved in Dulbecco's modified Eagle's medium (DMEM)/F-12. Enzymatic digestion was carried out for 1 h in a 37°C water bath, with manual shaking every 15 min. After digestion, isolated individual follicles were washed three times with culture medium and plated on glass-bottom 35-mm Petri dishes, coated with a thin layer of collagen gel. The collagen gel was prepared by spreading 8 µl of a collagen solution (3 mg/ml in 0.2% acetic acid) onto the glass surface, followed by addition of a neutralizing solution (0.4 M NaHCO_3_, 0.2 M HEPES [pH 7.4]). For the culture of individual thyroid cells, follicles isolated from two to three mice were seeded in a 100-mm culture dish and grown to confluence in a monolayer for 3–4 d. They were then completely dissociated to single cells with trypsin (0.25%)-EDTA (0.02%) and plated on 24-mm glass coverslips. Follicles and isolated thyroid cells were maintained in DMEM/F-12+20% FCS (37°C, 5% CO_2_).

FRTL5 cells were cultured in Coon's modified Ham's F12 medium supplemented with 5% FCS, 1% penicillin, 1% streptomycin, and a mixture of five hormones, and bTSH (6H) as previously described [89]. Twenty-four hours before the cell fractionation and adenylyl cyclase assay experiments, FRTL5 cells were switched to complete medium without bTSH (5H).

Primary mouse embryonic fibroblasts (MEFs) and cortical neurons were isolated from embryonic day (E) 14.5 transgenic embryos as previously described [14],[90]. Cortices were dissociated with trypsin and cells were plated onto poly-d-lysine-coated glass coverslips in the serum-free Neurobasal-A medium containing B-27 supplement, 0.5 mM l-glutamine, antibiotics, and 25 µM glutamate. Twenty-four hours later, the medium was changed to glutamate-free. Experiments were performed 2 d after plating. MEF isolation was performed using standard trypsin digestion of the embryos with subsequent plating on glass coverslips in DMEM medium containing 10% FCS, 2 mM l-glutamine, and antibiotics. Imaging experiments were performed 24 h after the isolation. Adult cardiac myocytes were isolated and measured 2–5 h after isolation as described [26]. Peritoneal macrophages were isolated as described [14] and maintained for 24 h in DMEM medium containing 10% FCS, 2 mM l-glutamine, and antibiotics. Experiments were performed 24–48 h after the isolation. Cos-7 and HEK293 cells were cultured in DMEM+10% FCS.

### TSH Labeling

TSH labeling was performed with Alexafluor488 or Alexafluor594 succinimidyl esters, following the manufacturer's protocol. Briefly, 5 mg of bTSH were dissolved in 0.5 ml of 0.1 M NaHCO_3_ buffer (pH 8.3). Then, 0.5 mg of either reactive dye dissolved in DMSO was added to the tube while vortexing, and the reaction was incubated for 1 h at room temperature with continuous stirring. The protein conjugates were separated from the unreacted dyes by gel filtration on Sephadex G25 columns. The concentration of TSH-Alexafluor488 and TSH-Alexafluor594 in the collected fractions was about 1 mg/ml. The degree of labeling was typically of one to two fluorescent moieties per molecule of TSH. After labeling, the TSH preparations were immediately aliquoted and stored at −20°C.

### Determination of cAMP Levels

Cos-7 cells were transfected with human TSH receptor cDNA or control empty vector by the diethylaminoethy-dextran method followed by a dimethylsulfoxide shock. Two days after transfection, the cells were used for cAMP determinations and flow immunocytofluorimetry to evaluate the transfection efficiency. For cAMP determinations, culture medium was replaced with Krebs-Ringer-HEPES buffer (KRH) for 30 min. Thereafter, the cells were incubated for 60 min in fresh KRH supplemented with 25 µM rolipram and various concentrations of labeled or unlabeled bTSH. At the end of the 1-h incubation, the medium was discarded and samples were extracted with 0.1 M HCl. The cell extracts were dried in a vacuum concentrator, resuspended in water, and diluted appropriately for cAMP evaluation by radioimmunoassay, utilizing standard procedures. cAMP levels in primary thyroid cells were measured with a commercial ELISA (CA200; Sigma-Aldrich), following the manufacturer's protocol.

### Evaluation of TSH-Alexa594 Specific Binding

HEK293 cells were plated on 24-mm round glass coverslips and transfected with human TSH receptor cDNA or control empty vector by Effectene, following the manufacturer's protocol. After 48 h, the medium was replaced with a buffer containing 144 mM NaCl, 5.4 mM KCl, 2 mM CaCl_2_, 1 mM MgCl_2_, 20 mM HEPES, 1% BSA (pH 7.3) and treated with 3 µg/ml TSH-Alexa594. In addition, the specific binding of TSH-Alexa594 was confirmed in primary mouse thyroid cells by competition with a 100-fold molar excess of unlabeled TSH (unpublished data). TSH-Alexa594 bound to cells was visualized by TIRF microscopy.

### FRET Measurements and Cell Imaging

For fluorescent microscopy, glass-bottom Petri dishes or glass coverslips mounted in an experimental chamber were placed on a Zeiss Axiovert 200 inverted microscope equipped with an oil-immersion 63× objective, a polychrome IV light source (Till Photonics), a 505 DCXR beam splitter, and a CoolSNAP-HQ CCD-camera (Visitron Systems). FRET was monitored using MetaFluor 5.0 software (Molecular Devices) as the ratio between emission at 535±20 nm (YFP) and emission at 480±15 nm (CFP), upon excitation at 436±10 nm. The imaging data were analyzed utilizing MetaMorph 5.0 (Molecular Devices) and Prism (GraphPad Software) software, by correcting for spillover of CFP into the 535-nm channel and direct YFP-excitation, to give corrected YFP/CFP ratio data. Images were acquired every 5 s, with 5-ms illumination time, which resulted in negligible photobleaching for over 30-min observation. To study agonist-induced changes in FRET, cells and thyroid follicles were continuously superfused with phenol red–free medium containing 1% BSA or the same plus agonists and/or endocytosis inhibitors, with a custom apparatus. All experiments were performed at 37°C. Confocal images were acquired with a Leica SP5 confocal microscope (Leica). To visualize TSH-Alexa594, fixed cells were excited with a 594-nm laser line, and images were acquired with a high-sensitivity APD detector. TIRF images were acquired with a Leica AM TIRF microscope, equipped with 488- and 561-nm laser lines and a fast high-sensitivity EM-CCD camera (Cascade 512B). Confocal and TIRF images were analyzed with ImageJ (U. S. National Institutes of Health, http://rsb.info.nih.gov/ij/, 1997–2007). AVI Videos were compressed with the Cinepak codec (Radius).

### Immunofluorescence and BODIPY-Forskolin Labeling

Cells were plated on 24-mm glass coverslips, washed with PBS and fixed with 4% paraformaldehyde for 15 min at room temperature. Cells were then permeabilized with PBS+0.1% Triton X-100 for 3 min at room temperature, blocked with PBS+5% goat serum for 1 h at room temperature, incubated with the indicated primary antibodies overnight at 4°C and incubated with the appropriate secondary antibodies for 2 h at room temperature. Antibodies against Gα_s_, adenylyl cyclase III, adenylyl cyclase V/VI, Golgi 58K, VASP, and P-VASP were used at 1∶50 dilution. The Rab7 antibody was used at 1∶200 dilution. All antibody solutions were prepared in PBS+5% goat serum. Secondary antibodies were used at 1∶400 (Cy2-conjugated goat anti-rabbit and anti-mouse polyclonal antibodies) or 1∶2,000 (Alexafluor594-conjugated goat anti-rabbit polyclonal antibody) dilutions. The specificity of the immunofluorescent stainings was evaluated by omitting the primary antibodies and by competition with the peptides used to raise the primary antibodies (for Gα_s_, adenylyl cyclase III, and adenylyl cyclase V/VI). In addition, the specificity of Gα_s_ immunofluorescence was evaluated by comparing the localization of transfected Gα_s_-YFP and Gα_s_ immunostaining in HEK293 cells (unpublished data). BODIPY-forskolin labeling was performed by incubating the cells for 10 min at room temperature with 100 nM BODIPY-forskolin dissolved in PBS+1% BSA. Then, the cells were washed twice with PBS and imaged immediately.

### Actin Staining

Cells were plated on 24-mm glass coverslips, washed with PBS, and then fixed with 4% paraformaldehyde for 10 min at room temperature. Cells were then permeabilized with PBS+0.1% Triton X-100 for 3 min at room temperature, blocked with PBS+1% BSA for 1 h at room temperature, and incubated with 1 U of Alexafluor488-conjugated phalloidin for 20 min at room temperature.

### Electron Microscopy

Primary mouse thyroid cells were fixed with 2% paraformaldehyde and 0.2% glutaraldehyde in PBS for 1 h, embedded in 12% gelatin, and infiltrated with 2.3 M sucrose. Ultrathin cryosections were obtained with a Reichert-Jung Ultracut E with a FC4E cryoattachment and collected on copper-formvar-carbon-coated grids. Immunogold labeling on ultrathin cryosections was performed as previously described [91]. Briefly, the sections were incubated with the rabbit anti-adenylyl cyclase III antibody or the rabbit anti-Alexafluor488 antibody, followed by incubation with 15-nm protein A-gold. Control sections were incubated with an unrelated antibody or without primary antibodies. Very low levels of labeling were detected in all control sections (unpublished data). All samples were examined with a Philips CM10 or a Fei TECNAI G12 electron microscope.

### Preparation of Concanavalin A Magnetic Beads

Streptavidin magnetic beads (Dynabeads Biotin Binder) were washed using a modification of the manufacturer's protocol. Briefly, 600 µl of magnetic beads were placed in a 1.5-ml Eppendorf test tube and were separated from the solution by placing the vial in contact with a magnet. The supernatant was discarded by pipetting, and the beads were washed with 1 ml of TBS buffer (150 mM NaCl, 50 mM Tris-HCl [pH 7.4]) supplemented with 2 mM EDTA and 1 mg/ml BSA, and resuspended in 600 µl of the same buffer. Then, 60 µl of biotinylated concanavalin A (5 mg/ml) were added, and the suspension was mixed in a rotor for 60 min at room temperature to allow binding of biotinylated concanavalin A to streptavidin. Finally, the beads were washed thrice with TBS buffer supplemented with 2 mM EDTA and 1 mg/ml BSA, and resuspended in 600 µl of homogenization buffer (HB: 250 mM sucrose, 25 mM KCl, 2.5 mM Mg(OAc)_2_, 25 mM Hepes/KOH [pH 7.4]) and stored at 4°C until use.

### Cell Fractionation

The protocol for plasma membrane separation with concanavalin A immobilized on magnetic beads was based on the method described by Lee et al. [47]. FRTL5 cells (20 100-mm Petri dishes per condition) were stimulated with 30 U/l bTSH for 30 min at 37°C where indicated, and harvested by trypsinization. The subsequent steps were performed at 4°C. First, FRTL5 cells were washed once with 5H medium and twice with TBS, and resuspended in HB. Then, the equivalent of 100 µl of concanavalin A beads for each Petri dish was added to the cell suspension, and the samples were incubated for 30 min at 37°C under continuous rotation. At the end of the first incubation, the cells were lysed by gently passing them eight times through a 1-ml syringe with a 26G needle, and the plasma membrane fraction bound to the beads was separated with the help of a magnet. Thereafter, the nuclei were sedimented by centrifuging the samples at 800×*g* for 10 min at 4°C. The remaining postnuclear supernatant was further purified by adding 100 µl of concanavalin A beads for each Petri dish and repeating the incubation and the magnetic separation procedure to give rise to a second bead-bound fraction and to the final intracellular fraction. The beads with the attached fractions were washed twice with HB supplemented with 1 mg/ml BSA and twice with HB. For Western blot experiments, the proteins bound to the magnetic beads were eluted by resuspending the beads in SDS sample buffer (2% SDS, 10% glycerol, 50 mM dithiothreitol, 0.01% bromophenol blue, 62.5 mM Tris/HCl [pH 6.8]), incubating them at 60°C for 10 min and removing the beads with the help of a magnet. For adenylyl cyclase activity determinations, the beads with the attached membrane fraction were resuspended in HB buffer and used directly in the adenylyl cyclase assay.

### Adenylyl Cyclase Assay

The determination of adenylyl cyclase activity was based on the method originally described by Jakobs et al. [92],[93]. Briefly, 300 µg of proteins were added to an incubation medium in a total volume of 100 µl with final concentrations of 50 mM Tris/HCl (pH 7.4), 300 mM sucrose, 100 µM cAMP, 10 µM GTP, 100 µM ATP, 1.25 mM Mg(Ac)_2_, 100 µM IBMX, 0.2% BSA, 15 mM creatine phosphate, and 0.4 mg/ml creatine kinase. Samples were incubated with about 300,000 cpm of [α-^32^P]-ATP for 60 min in the incubation medium. The reaction was stopped by addition of 400 µl of a 125 mM ZnAc solution and 500 µl of a 144 mM Na_2_CO_3_ solution. Samples were centrifuged for 5 min at 14,000 rpm in a laboratory microcentrifuge. Finally, 800 µl of the resulting supernatant were applied to alumina WN-6 columns that were eluted twice with 2 ml of 100 mM Tris/HCl (pH 7.4). The eluates were counted in a β-counter.

### Analysis of VASP Phosphorylation

Primary mouse thyroid cells were seeded in six-well plates and starved in serum-free medium for 4 h. Thereafter, the cells were preincubated in serum-free medium plus/minus endocytosis inhibitors and incubated at 37°C in the presence of 1 U/l bTSH for 30 min. This concentration of TSH was chosen because it elicited a robust phosphorylation of VASP, without causing the VASP signal to be completely saturated. At the end of the incubation, the cells were washed with PBS, lysed with SDS sample buffer, and immediately heated for 5 min at 95°C. The levels of VASP phosphorylation were evaluated by Western blot analysis. Afterwards, the membranes were stripped and reprobed with an antibody against total VASP.

### Western Blot Analysis

Protein concentration was determined by BCA assay. Protein extracts were electrophoresed on a 10% SDS polyacrylamide gel and electro-transferred to a nitrocellulose membrane. Membranes were blocked with TBS-T+3% milk, probed with the indicated primary antibody overnight at 4°C, and incubated with the appropriate horseradish peroxidase-conjugated secondary antibody for 1 h at room temperature. The following dilutions of primary antibodies were used: anti-Na^+^/K^+^ ATPase 1∶10,000, anti-EEA1 1∶400, anti-Golgi 58K 1∶5,000, anti-Gα_s_ 1∶10,000, and anti-adenylyl cyclase III 1∶10,000. Detection was performed utilizing the ECL detection kit.

### Simulations

Simulations were performed in the Virtual Cell modeling environment [48]–[50]. The receptor was placed on the plasma membrane. G-proteins and adenylyl cyclase were both on the plasma membrane and on the membrane of the ICSC. ATP, cAMP, PDE4, and PKA were cytosolic. In some instances, we simulated the internalization of the receptor and its ligand to the ICSC. We used the initial concentrations and diffusion coefficients utilized by Neves et al. [21], which are mostly based on experimentally determined values. Initial concentrations, displayed in units of molecules/µm^2^ for membrane components and µM for cytosolic components, are provided in Table S1. For those components not shown, the initial concentration was set at zero. Reactions and kinetic parameters are shown in Table S2. Spatial simulations were run using the regular grid, finite volume solver. The geometric parameters used in the simulations are provided in Table S3. The mean steady-state concentration of cAMP, obtained by running the model in the absence of ligand until all the components reached steady state, was used as the initial concentration for subsequent simulations. A detailed description of the mathematical model and the results of additional simulations can be found in Text S1. The entire model, parameters and geometries are available at http://vcell.org/.

### Statistical Analysis

Values are expressed as mean±standard error of the mean (SEM). Data normality was checked with the Kolmogorov-Smirnov test. Differences between means were assessed by two-tailed *t*-test (for two groups) or one-way ANOVA followed by Bonferroni post hoc test (for three or more groups). For analysis of signal normalization after TSH washout, signal reversibility was calculated from YFP/CFP ratio values by setting the minimum value equal to zero and the value before TSH stimulation equal to 100%. The values obtained from different replicates were globally fitted to a first-order exponential function. Fits were compared by using F test, having a null hypothesis that *Y*_max_ values were the same for all datasets. Alternatively, data from each replicate were individually fit to a first-order exponential function, and the obtained *Y*_max_ values were compared by one-way ANOVA.

## Supporting Information

Figure S1 **Comparison of cAMP levels in thyroid cells isolated from wild-type and transgenic mice.** Primary thyroid cells isolated from either wild-type or CAG-Epac1-camps mice were starved overnight in medium without serum and stimulated for 60 min with different concentrations of bTSH in the presence of 750 µM IBMX. cAMP levels were determined with an immunoenzymatic assay. Three biological replicates per condition were used. Error bars indicate SEM.
(0.03 MB TIF)

Figure S2 **cAMP reversibility after stimulation with a forskolin analog.** Primary mouse thyroid follicles were treated with 7-deacetyl-7-[O-(N-methylpiperazino)-γ-butyryl)]-forskolin (DMPB-forskolin), a forskolin analog with improved water solubility. Reported is a representative trace from a thyroid follicle that was initially stimulated for 3 min and later on for 10 min. Note that DMPB-forskolin produced cAMP increases comparable to those obtained with TSH (for comparison, see Figure 5B–5D). In contrast to what was observed with TSH, the signals produced by DMPB-forskolin were completely reversible upon washout. Data are representative of ten independent experiments.
(0.04 MB TIF)

Figure S3 **Evaluation of the biological activity of TSH-Alexa594.** (A) Cos-7 cells were transfected with TSH receptor cDNA and stimulated 48 h later with various concentrations of either unlabeled TSH or TSH labeled with Alexafluor594. The graph shows the levels of intracellular cAMP, measured by a radioimmunoassay. Eight replicates for each point were used. Error bars indicate SEM. (B) Binding of TSH-Alexa594 to HEK293 cells expressing the TSH receptor. HEK293 cells were transfected with either human TSH receptor cDNA or the empty expression vector (control). Forty-eight hours after transfection, the cells were stimulated with 3 µg/ml TSH-Alexa594 and visualized by TIRF microscopy. The images were acquired 10 min after addition of the fluorescent ligand. They are representative of 18–20 cells per condition analyzed in three independent experiments.
(0.54 MB TIF)

Figure S4 **Fluorescent TSH internalization in whole thyroid follicles.** Primary thyroid follicles obtained from CAG-Epac1-camps mice were stimulated with 3 µg/ml TSH-Alexa594 for 60 min, fixed, and then visualized by confocal microscopy. To isolate the Alexa594 signal, the background autofluorescence was subtracted from the image by spectral unmixing. To this end, an additional reference image was acquired at 405-nm excitation and 425–450-nm emission. This reference image was multiplied by a correction factor (calculated from the relative intensities of Alexa594 and of reference images of unlabeled thyroid follicles) and subtracted from the Alexa594 image. Shown is the corrected Alexa594 image. Images are representative of 15 follicles visualized in three independent experiments.
(0.64 MB TIF)

Figure S5 **Subcellular localization of Gα_s_ and adenylyl cyclases in primary mouse thyroid cells.** Cells were fixed with 4% paraformaldehyde and stained with primary antibodies against Gα_s_, adenylyl cyclase III, or adenylyl cyclase V/VI. Shown are low-magnification images acquired with a laser-scanning confocal microscope. Images are representative of five independent experiments.
(2.65 MB TIF)

Figure S6 **Specificity of immunofluorescence for Gα_s_ and adenylyl cyclases.** The specificity of Gα_s_, adenylyl cyclase III, and adenylyl cyclase V/VI immunofluorescent stainings was evaluated by preincubating the primary antibodies with a 5-fold (by weight) excess of blocking peptides, followed by the standard immunofluorescence procedure. Images are representative of three independent experiments.
(2.90 MB TIF)

Figure S7 **Immunogold labeling of internalized TSH (A–C) and adenylyl cyclase III (D and E).** Primary mouse thyroid cells were stimulated with normal medium (A and D) or medium+3 µg/ml TSH-Alexa488 (B, C, and E) for 30 min. Representative images of the intracellular localization of TSH-Alexa488 and adenylyl cyclase III are shown. (A–C) Immunogold labeling with an antibody against Alexa488. No staining was observed in cells that were not stimulated with TSH-Alexa488 (A). By contrast, in cells that were stimulated with TSH-Alexa488 for 30 min, a positive immunogold staining was present in early and late endosomes (B), as well as in denser vesicles probably representing a degradative compartment (C). (D and E) Immunogold labeling with an antibody against adenylyl cyclase III. Adenylyl cyclase III was found occasionally on the plasma membrane and on small subplasmalemmal vesicles (unpublished data), in the Golgi area (D) and on endosomal membranes (E). Stimulation with TSH-Alexa488 appeared to just slightly modify the distribution of adenylyl cyclase III, with a tendency towards a reduction in the Golgi area and an increase in the endosomal compartment. Images are representative of three independent experiments. Bars indicate (A) 0.16 µm, (B) 0.48 µm, (C) 0.17 µm, (D) 0.25 µm, and (E) 0.23 µm.
(2.26 MB TIF)

Figure S8 **Effect of pH on TSH receptor-cAMP signaling.** Primary mouse thyroid follicles isolated from CAG-Epac1-camps mice were preincubated for 20 min in medium adjusted to the indicated pH values and then visualized by time-lapse fluorescence microscopy. The cAMP response to TSH stimulation was monitored as described above. Traces are representative of six to eight independent experiments per condition.
(0.08 MB TIF)

Figure S9 **Effect of varying the adenylyl cyclase density on the ICSC membrane.**
(0.09 MB TIF)

Figure S10 **Effect of different ICSC geometries.**
(0.27 MB TIF)

Figure S11 **Effect of GPCR recycling.**
(0.38 MB TIF)

Figure S12 **Western blot analysis of VASP phosphorylation.** Primary mouse thyroid cells were preincubated with normal medium (c), medium plus 0.43 M sucrose (s) for 10 min, or medium plus 80 µM dynasore (d) for 20 min. Cells were then stimulated with 1 U/l TSH for 30 min, in the presence or absence of endocytosis inhibitors as indicated. Levels of P-VASP (Ser 157) and total VASP were evaluated by Western blot analysis. The experiment was performed three times with similar results. Shown are the results of a representative experiment.
(0.10 MB TIF)

Table S1 **Initial concentrations of components used in the model.**
(0.07 MB PDF)

Table S2 **Reactions and kinetic parameters used in the model.**
(0.07 MB PDF)

Table S3 **Geometric parameters used in the model.**
(0.71 MB PDF)

Text S1 **Detailed description of the mathematical model.**
(0.13 MB PDF)

Video S1 **3-D reconstruction of a primary mouse thyroid follicle isolated form CAG-Epac1-camps mice.** A stack of YFP images on the *z*-axis was acquired with a laser-scanning confocal microscope. The 3-D reconstruction was performed with ImageJ software. The video shows a 360° rotation on the *y*-axis.
(1.07 MB AVI)

Video S2 **Sequence of YFP/CFP ratio images of a thyroid follicle stimulated with 100 U/l TSH and 10 µM forskolin.** The video shows the entire sequence from which the frames reported in Figure 4A were derived. Time between frames was 5 s. Playback is accelerated (ten frames/second).
(3.71 MB AVI)

Video S3 **Dynamic visualization of internalized TSH-Alexa594.** Primary mouse thyroid cells were stimulated with 3 µg/ml TSH-Alexa594 for 20 min. After a rapid wash with fresh medium, TSH-Alexa594 fluorescence was visualized with a TIRF microscope set to have a high penetration depth. The video shows the entire sequence from which the frames reported in Figure 9 were derived. Time between frames was 1 s. Playback is accelerated (ten frames/second).
(5.81 MB AVI)

Video S4 **A 360° rotation on the** ***x*** **-axis of the 3-D reconstruction in** **Figure 11A** **.**
(0.35 MB AVI)

Video S5 **A 360° rotation on the** ***x*** **-axis of the 3-D reconstruction in** **Figure 11C** **.**
(0.26 MB AVI)

Video S6 **Dynamic visualization of internalized TSH-Alexa594 and adenylyl cyclases.** Primary mouse thyroid cells were stimulated with 3 µg/ml TSH-Alexa594 for 20 min, followed by staining with BODIPY-forskolin. TSH-Alexa594 fluorescence was visualized with a TIRF microscope set to have a high penetration depth. Data are representative of 20 cells analyzed in three independent experiments. Time between frames was 10 s. Playback is accelerated (four frames/second).
(0.16 MB AVI)

Video S7 **Effect of hypertonic sucrose on TSH-Alexa594 internalization.** Primary mouse thyroid cells were preincubated with 0.43 M sucrose for 10 min and stimulated with 3 µg/ml TSH-Alexa 594 for 20 min in the presence of 0.43 M sucrose. After a rapid wash with fresh medium, TSH-Alexa594 fluorescence was visualized with a TIRF microscope set to have a high penetration depth. Data are representative of 18 cells analyzed in three independent experiments. Time between frames was 10 s. Playback is accelerated (one frame/second).
(0.85 MB AVI)

Video S8 **Control for** **Videos S7** **and** **S9** **.** Primary mouse thyroid cells were stimulated with 3 µg/ml TSH-Alexa594 in the absence of endocytosis inhibitors. After a rapid wash with fresh medium, TSH-Alexa594 fluorescence was visualized with a TIRF microscope set to have a high penetration depth. Data are representative of 21 cells analyzed in three independent experiments. Time between frames was 10 s. Playback is accelerated (one frame/second).
(1.38 MB AVI)

Video S9 **Effect of dynasore on TSH-Alexa594 internalization.** Primary mouse thyroid cells were preincubated with 80 µM dynasore for 20 min and stimulated with 3 µg/ml TSH-Alexa594 for 20 min, in the presence of 80 µM dynasore. After a rapid wash with fresh medium, TSH-Alexa594 fluorescence was visualized with a TIRF microscope set to have a high penetration depth. Data are representative of 25 cells analyzed in three independent experiments. Time between frames was 10 s. Playback is accelerated (one frame/second).
(0.51 MB AVI)

## Abbreviations

FRET: fluorescence resonance energy transfer
ICSC: intracellular signaling compartment
