# Supplementary figures and images for: Persistent cAMP-Signals Triggered by Internalized G-Protein–Coupled Receptors
Source: PLoS Biol. 2009 Aug 18;7(8):e1000172. doi: 10.1371/journal.pbio.1000172 (PMC2718703; doi:10.1371/journal.pbio.1000172)

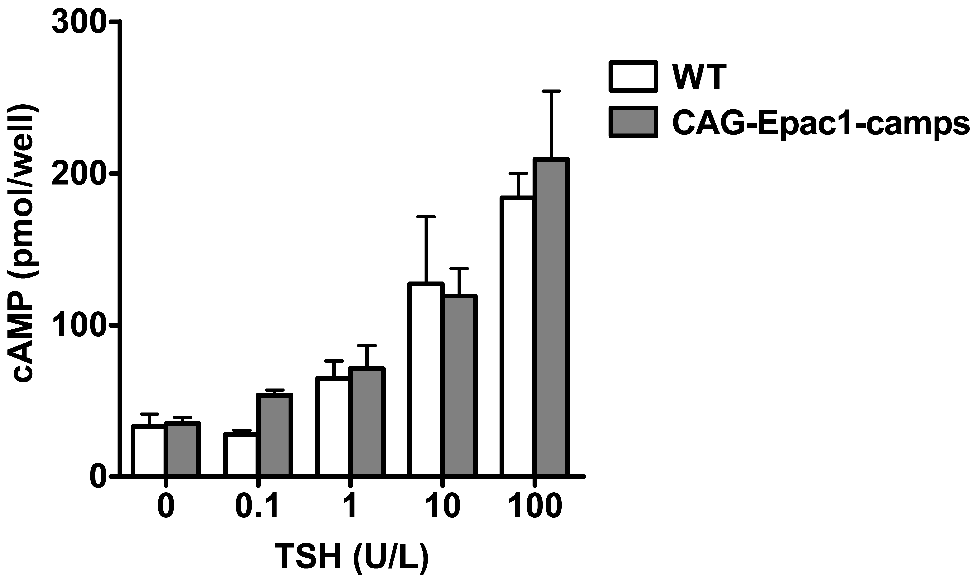

Supplement: Figure S1 — Comparison of cAMP levels in thyroid cells isolated from wild-type and transgenic mice. Primary thyroid cells isolated from either wild-type or CAG-Epac1-camps mice were starved overnight in medium without serum and stimulated for 60 min with different concentrations of bTSH in the presence of 750 µM IBMX. cAMP levels were determined with an immunoenzymatic assay. Three biological replicates per condition were used. Error bars indicate SEM. (0.03 MB TIF) [file pbio.1000172.s001.tif]

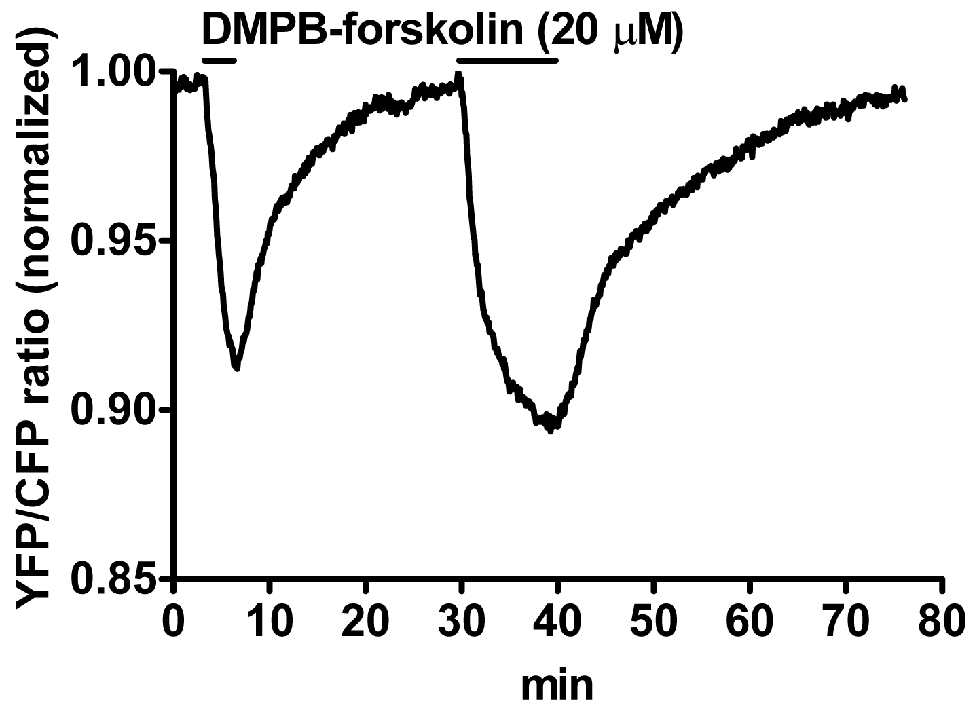

Supplement: Figure S2 — cAMP reversibility after stimulation with a forskolin analog. Primary mouse thyroid follicles were treated with 7-deacetyl-7-[O-(N-methylpiperazino)-γ-butyryl)]-forskolin (DMPB-forskolin), a forskolin analog with improved water solubility. Reported is a representative trace from a thyroid follicle that was initially stimulated for 3 min and later on for 10 min. Note that DMPB-forskolin produced cAMP increases comparable to those obtained with TSH (for comparison, see Figure 5B–5D). In contrast to what was observed with TSH, the signals produced by DMPB-forskolin were completely reversible upon washout. Data are representative of ten independent experiments. (0.04 MB TIF) [file pbio.1000172.s002.tif]

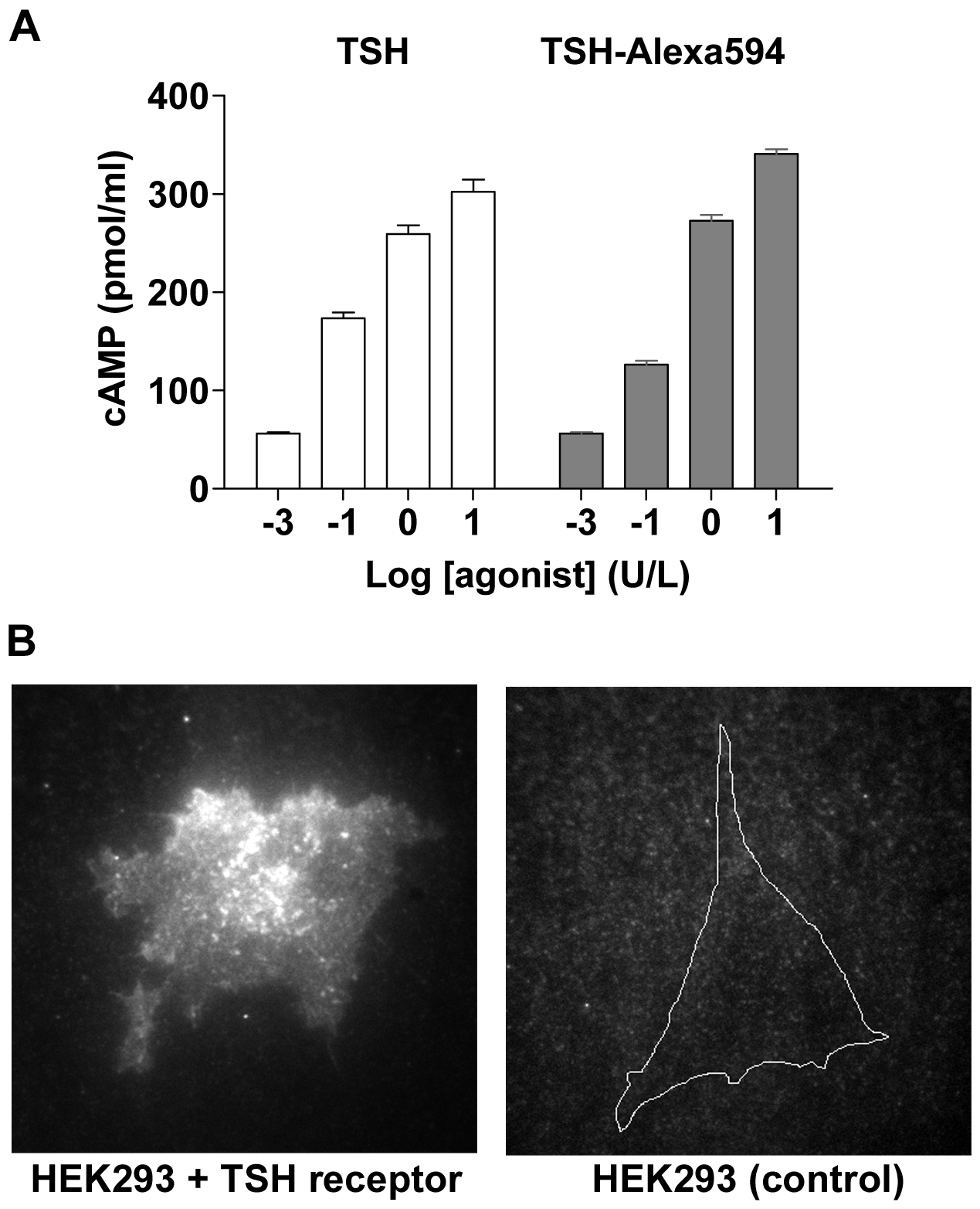

Supplement: Figure S3 — Evaluation of the biological activity of TSH-Alexa594. (A) Cos-7 cells were transfected with TSH receptor cDNA and stimulated 48 h later with various concentrations of either unlabeled TSH or TSH labeled with Alexafluor594. The graph shows the levels of intracellular cAMP, measured by a radioimmunoassay. Eight replicates for each point were used. Error bars indicate SEM. (B) Binding of TSH-Alexa594 to HEK293 cells expressing the TSH receptor. HEK293 cells were transfected with either human TSH receptor cDNA or the empty expression vector (control). Forty-eight hours after transfection, the cells were stimulated with 3 µg/ml TSH-Alexa594 and visualized by TIRF microscopy. The images were acquired 10 min after addition of the fluorescent ligand. They are representative of 18–20 cells per condition analyzed in three independent experiments. (0.54 MB TIF) [file pbio.1000172.s003.tif]

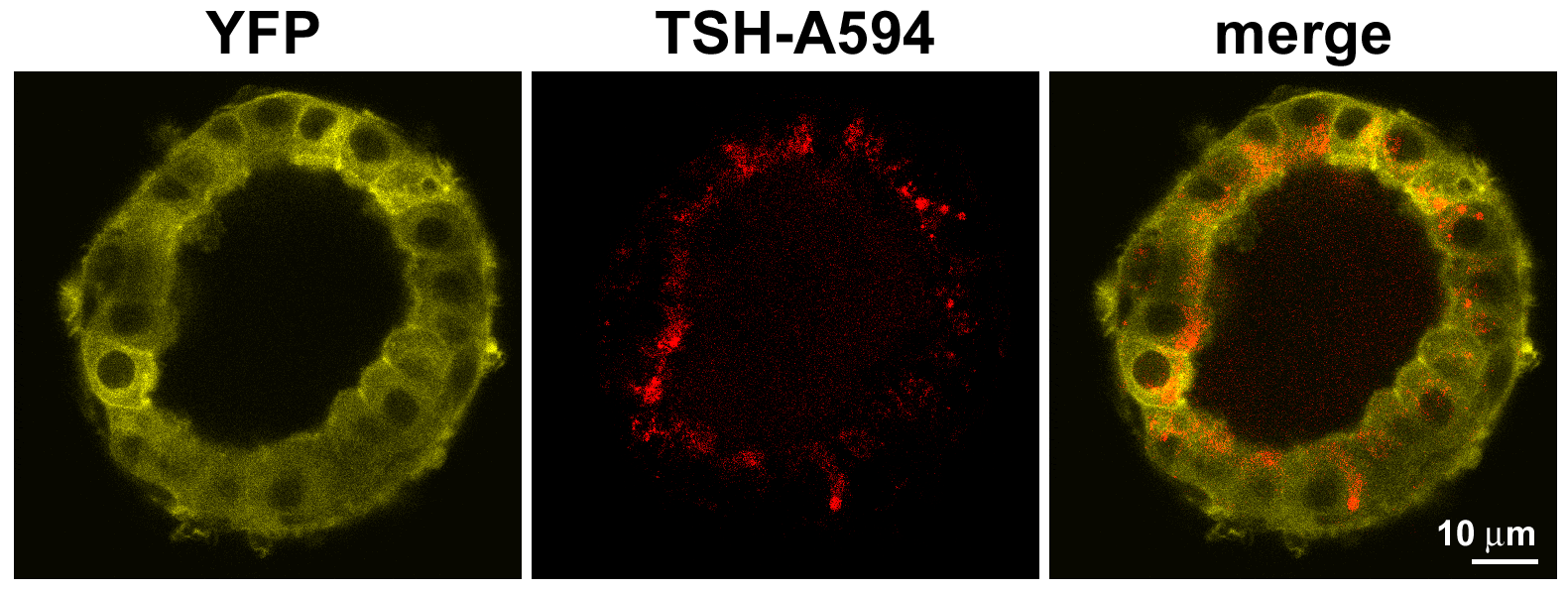

Supplement: Figure S4 — Fluorescent TSH internalization in whole thyroid follicles. Primary thyroid follicles obtained from CAG-Epac1-camps mice were stimulated with 3 µg/ml TSH-Alexa594 for 60 min, fixed, and then visualized by confocal microscopy. To isolate the Alexa594 signal, the background autofluorescence was subtracted from the image by spectral unmixing. To this end, an additional reference image was acquired at 405-nm excitation and 425–450-nm emission. This reference image was multiplied by a correction factor (calculated from the relative intensities of Alexa594 and of reference images of unlabeled thyroid follicles) and subtracted from the Alexa594 image. Shown is the corrected Alexa594 image. Images are representative of 15 follicles visualized in three independent experiments. (0.64 MB TIF) [file pbio.1000172.s004.tif]

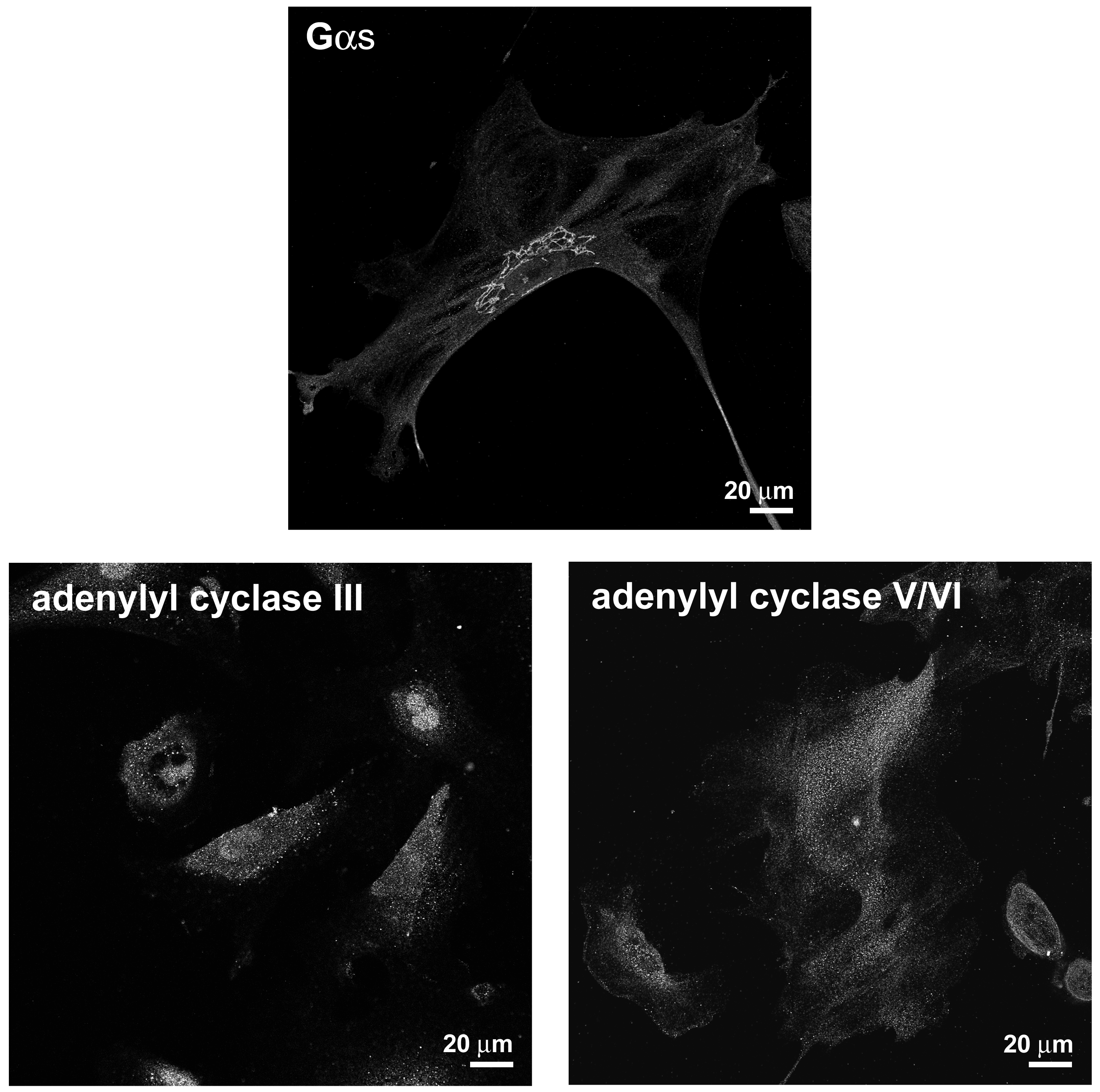

Supplement: Figure S5 — Subcellular localization of Gαs and adenylyl cyclases in primary mouse thyroid cells. Cells were fixed with 4% paraformaldehyde and stained with primary antibodies against Gαs, adenylyl cyclase III, or adenylyl cyclase V/VI. Shown are low-magnification images acquired with a laser-scanning confocal microscope. Images are representative of five independent experiments. (2.65 MB TIF) [file pbio.1000172.s005.tif]

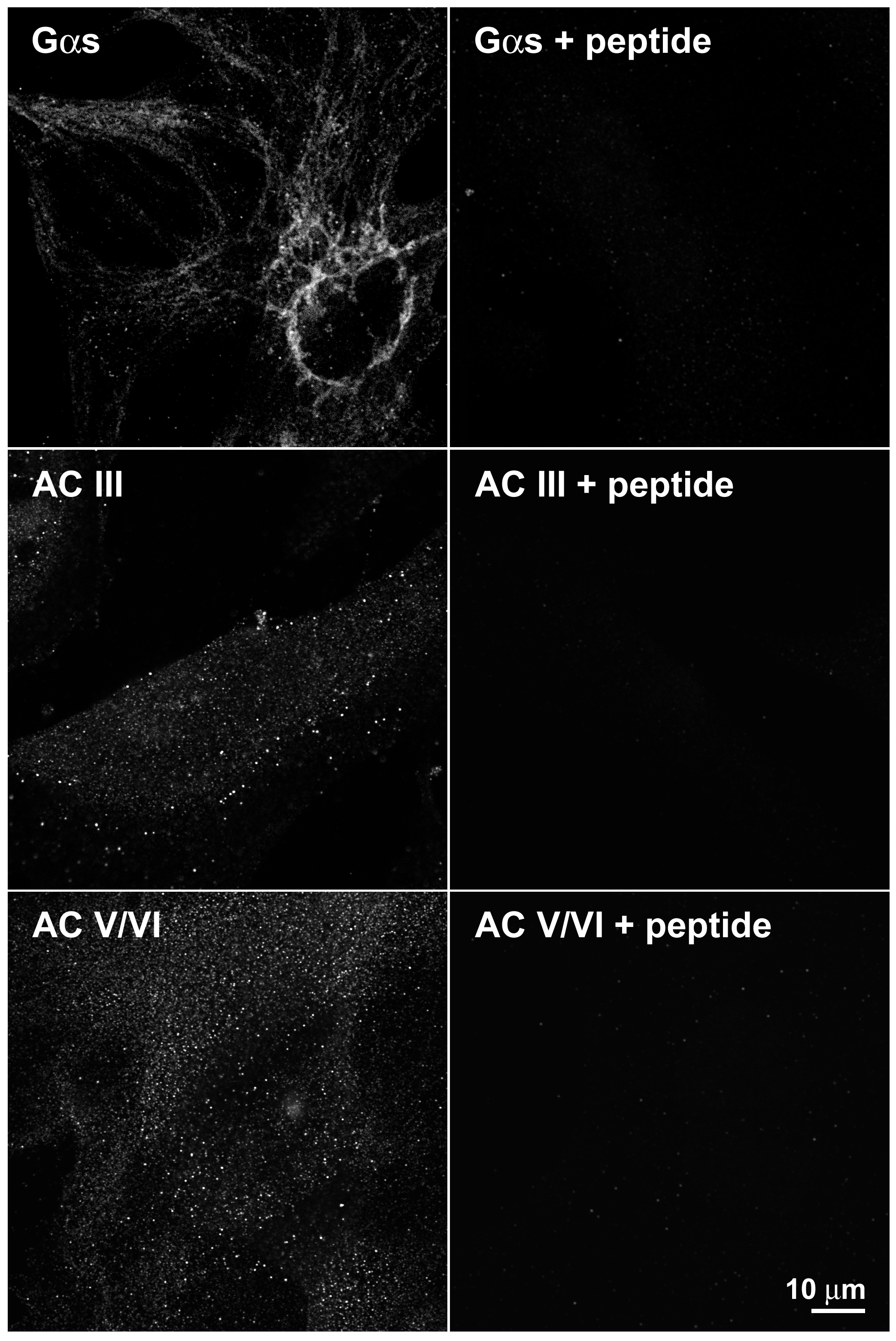

Supplement: Figure S6 — Specificity of immunofluorescence for Gαs and adenylyl cyclases. The specificity of Gαs, adenylyl cyclase III, and adenylyl cyclase V/VI immunofluorescent stainings was evaluated by preincubating the primary antibodies with a 5-fold (by weight) excess of blocking peptides, followed by the standard immunofluorescence procedure. Images are representative of three independent experiments. (2.90 MB TIF) [file pbio.1000172.s006.tif]

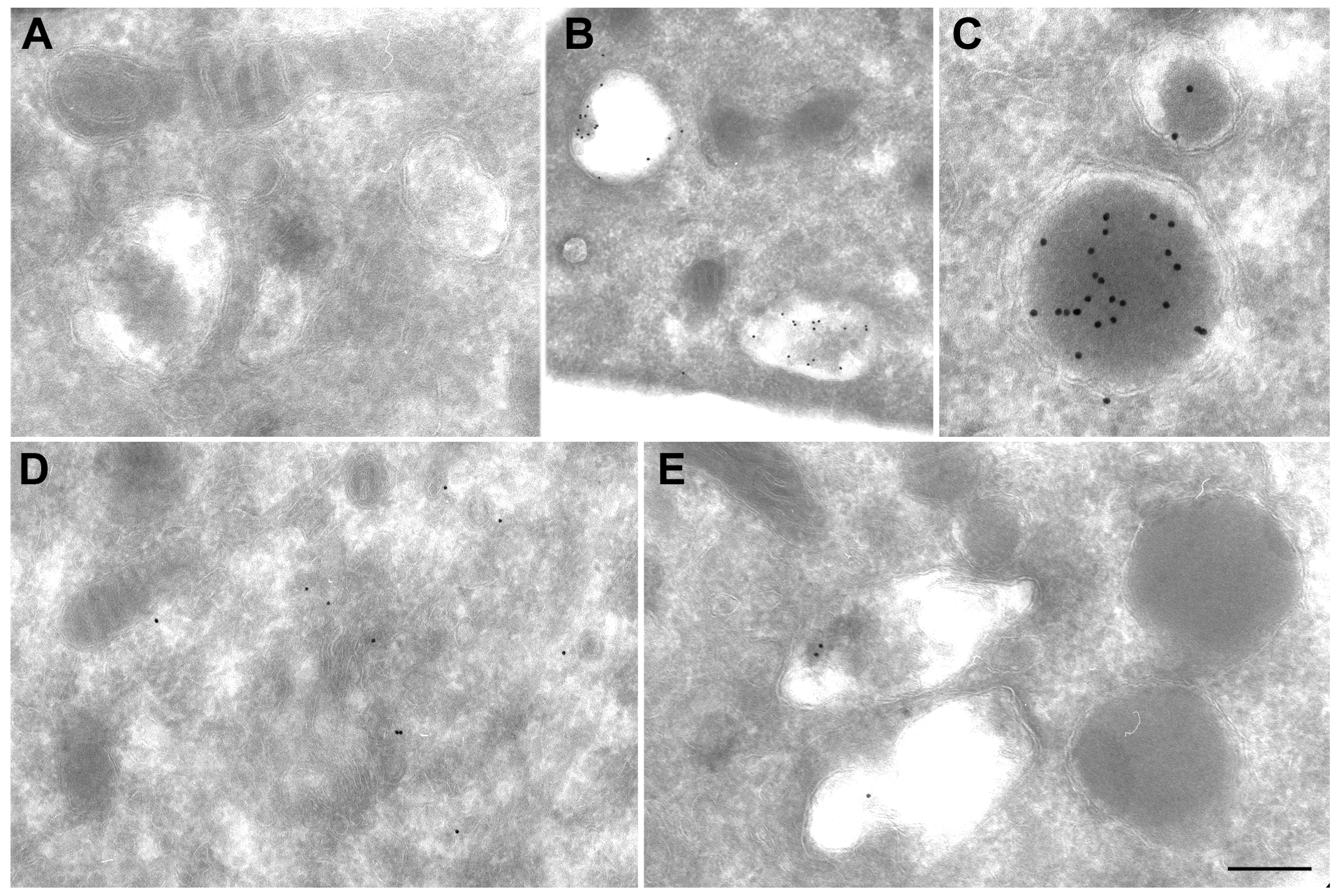

Supplement: Figure S7 — Immunogold labeling of internalized TSH (A–C) and adenylyl cyclase III (D and E). Primary mouse thyroid cells were stimulated with normal medium (A and D) or medium+3 µg/ml TSH-Alexa488 (B, C, and E) for 30 min. Representative images of the intracellular localization of TSH-Alexa488 and adenylyl cyclase III are shown. (A–C) Immunogold labeling with an antibody against Alexa488. No staining was observed in cells that were not stimulated with TSH-Alexa488 (A). By contrast, in cells that were stimulated with TSH-Alexa488 for 30 min, a positive immunogold staining was present in early and late endosomes (B), as well as in denser vesicles probably representing a degradative compartment (C). (D and E) Immunogold labeling with an antibody against adenylyl cyclase III. Adenylyl cyclase III was found occasionally on the plasma membrane and on small subplasmalemmal vesicles (unpublished data), in the Golgi area (D) and on endosomal membranes (E). Stimulation with TSH-Alexa488 appeared to just slightly modify the distribution of adenylyl cyclase III, with a tendency towards a reduction in the Golgi area and an increase in the endosomal compartment. Images are representative of three independent experiments. Bars indicate (A) 0.16 µm, (B) 0.48 µm, (C) 0.17 µm, (D) 0.25 µm, and (E) 0.23 µm. (2.26 MB TIF) [file pbio.1000172.s007.tif]

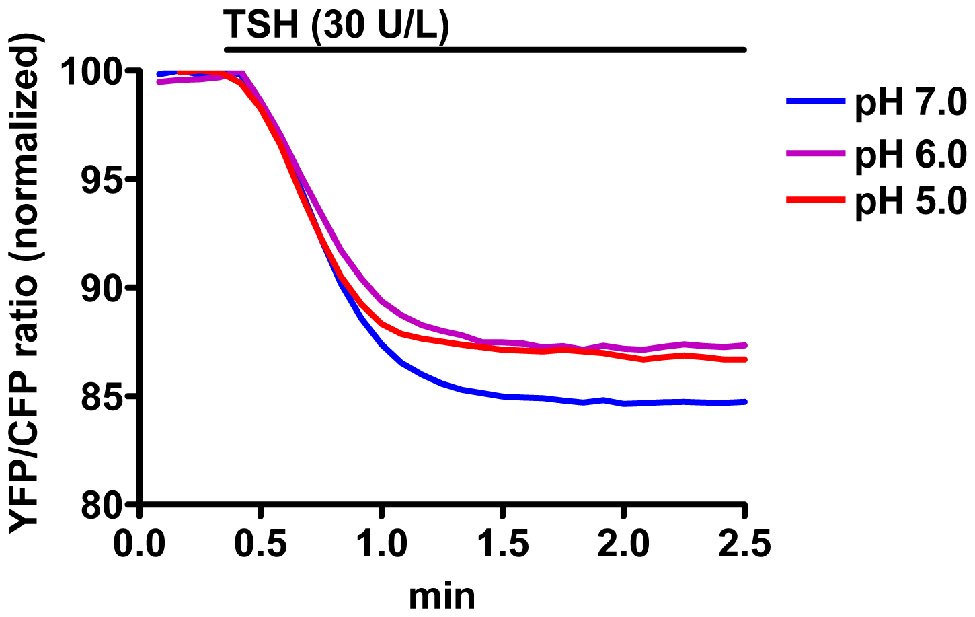

Supplement: Figure S8 — Effect of pH on TSH receptor-cAMP signaling. Primary mouse thyroid follicles isolated from CAG-Epac1-camps mice were preincubated for 20 min in medium adjusted to the indicated pH values and then visualized by time-lapse fluorescence microscopy. The cAMP response to TSH stimulation was monitored as described above. Traces are representative of six to eight independent experiments per condition. (0.08 MB TIF) [file pbio.1000172.s008.tif]

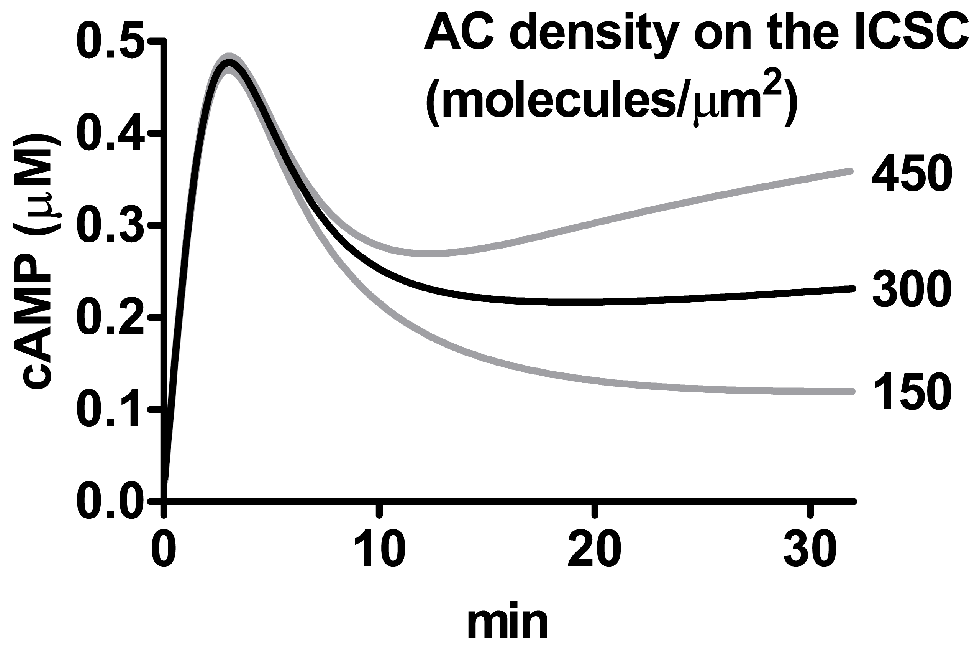

Supplement: Figure S9 — Effect of varying the adenylyl cyclase density on the ICSC membrane. (0.09 MB TIF) [file pbio.1000172.s009.tif]

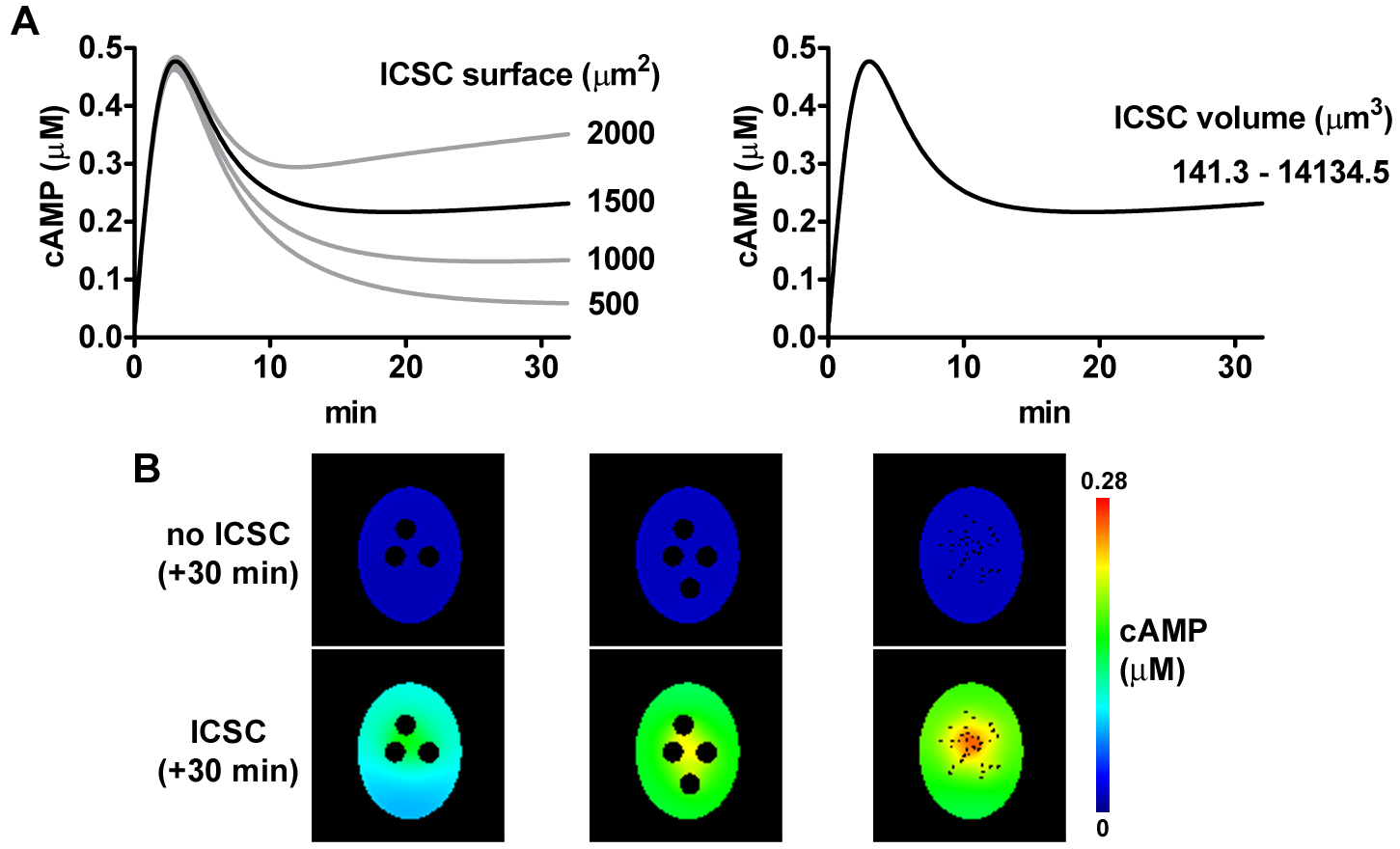

Supplement: Figure S10 — Effect of different ICSC geometries. (0.27 MB TIF) [file pbio.1000172.s010.tif]

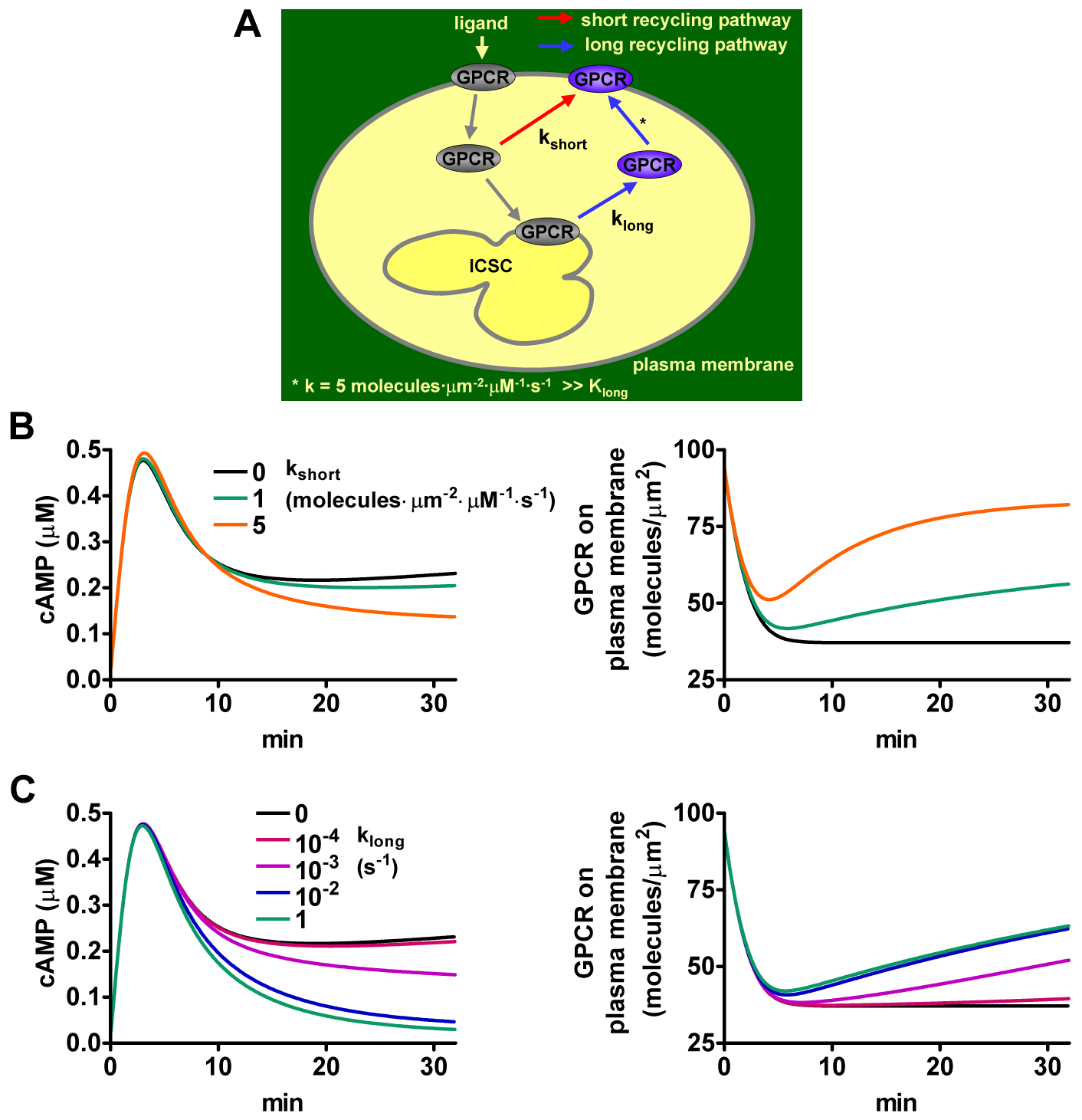

Supplement: Figure S11 — Effect of GPCR recycling. (0.38 MB TIF) [file pbio.1000172.s011.tif]

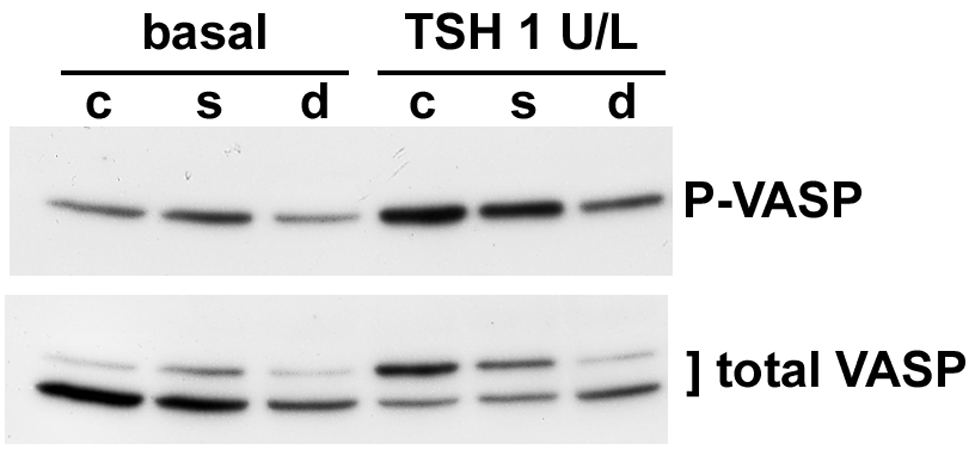

Supplement: Figure S12 — Western blot analysis of VASP phosphorylation. Primary mouse thyroid cells were preincubated with normal medium (c), medium plus 0.43 M sucrose (s) for 10 min, or medium plus 80 µM dynasore (d) for 20 min. Cells were then stimulated with 1 U/l TSH for 30 min, in the presence or absence of endocytosis inhibitors as indicated. Levels of P-VASP (Ser 157) and total VASP were evaluated by Western blot analysis. The experiment was performed three times with similar results. Shown are the results of a representative experiment. (0.10 MB TIF) [file pbio.1000172.s012.tif]
