# Supplementary material for: Persistent cAMP-Signals Triggered by Internalized G-Protein–Coupled Receptors
Source: PLoS Biol. 2009 Aug 18;7(8):e1000172. doi: 10.1371/journal.pbio.1000172 (PMC2718703; doi:10.1371/journal.pbio.1000172)
Supplement: Table S1 — Initial concentrations of components used in the model. (0.07 MB PDF) [file pbio.1000172.s013.pdf]

Table S1. Initial concentrations of components used in the model.

Component	Compartment	Concentration	Units	Diffusion coefficient ($\mu m^2/s$)
ligand	extracellular	1.0	μM	300.0
GPCR	plasma_membrane	94.0	molecules/μm^2	-
AC	plasma_membrane	300.0	molecules/μm^2	-
G_protein	cytoplasm	3.6	μM	6.0
GRK	cytoplasm	0.001	μM	6.0
cAMP	cytoplasm	0.009-0.041	μM	300.0
ATP	cytoplasm	5000.0	μM	300.0
PKA (R2C2)	cytoplasm	0.2	μM	-
PDE4	cytoplasm	0.2	μM	5.0
AMP	cytoplasm	1000.0	μM	300.0
AC	ICSC_membrane	300.0	molecules/μm^2	-
