# Supplementary material for: Persistent cAMP-Signals Triggered by Internalized G-Protein–Coupled Receptors
Source: PLoS Biol. 2009 Aug 18;7(8):e1000172. doi: 10.1371/journal.pbio.1000172 (PMC2718703; doi:10.1371/journal.pbio.1000172)
Supplement: Table S2 — Reactions and kinetic parameters used in the model. (0.07 MB PDF) [file pbio.1000172.s014.pdf]

Table S2. Reactions and kinetic parameters used in the model.

	Reaction	Parameters		
**plasma_membrane/ICSC_membrane:**				
1	ligand + GPCR <=> ligand_GPCR	Kf	1.0	$\mu M^{-1}.s^{-1}$
		Kr	0.2	s^{-1}
2	G_protein + GPCR <=> G_GPCR	Kf	0.3	$\mu M^{-1}.s^{-1}$
		Kr	0.1	s^{-1}
3	ligand + G_GPCR <=> ligand_G_GPCR	Kf	1.0	$\mu M^{-1}.s^{-1}$
		Kr	0.062	s^{-1}
4	ligand_GPCR + G_protein <=> ligand_G_GPCR	Kf	10.0	$\mu M^{-1}.s^{-1}$
		Kr	0.1	s^{-1}
5	ligand_G_GPCR <=> ligand_GPCR + Gs_GTP + bg	Kf	0.025	s^{-1}
		Kr	0	
6	ligand_GPCR + GRK -> ligand_GPCR_p	Km	15.0	molecules.μm^{-2}
		Kcat	0.104	s^{-1}
7	ligand_GPCR + GRK_bg -> ligand_GPCR_p	Km	4.0	molecules.μm^{-2}
		Kcat	1.34	s^{-1}
8	ATP + AC -> cAMP	Km	1030.0	μM
		Kcat	0.2	s^{-1}
9	AC + Gs_GTP <=> AC_active	Kf	500.0	$\mu M^{-1}.s^{-1}$
		Kr	1.0	s^{-1}
10	ATP + AC_active -> cAMP	Km	315.0	μM
		Kcat	8.5	s^{-1}
**cytoplasm:**				
11	Gs_GTP <=> Gs_GDP	Kf	0.067	μM^{-1}
		Kr	0	
12	Gs_GDP + bg <=> G_protein	Kf	6.0	$\mu M^{-1}.s^{-1}$
		Kr	0	
13	GRK + bg <=> GRK_bg	Kf	1.0	$\mu M^{-1}.s^{-1}$
		Kr	0.25	s^{-1}
14	cAMP + R2C2 <=> cAMP_R2C2	Kf	0.0059	$\mu M^{-1}.s^{-1}$
		Kr	0.0003	s^{-1}
15	cAMP + cAMP_R2C2 <=> cAMP2_R2C2	Kf	0.0059	$\mu M^{-1}.s^{-1}$
		Kr	0.0003	s^{-1}
16	cAMP + cAMP2_R2C2 <=> cAMP3_R2C2	Kf	8.35	$\mu M^{-1}.s^{-1}$
		Kr	0.0167	s^{-1}
17	cAMP + cAMP3_R2C2 <=> PKA_active	Kf	8.35	$\mu M^{-1}.s^{-1}$
		Kr	0.0167	s^{-1}
18	cAMP + PDE4 -> AMP	Km	1.3	μM
		Kcat	8.0	s^{-1}
