# Supplementary material for: Persistent cAMP-Signals Triggered by Internalized G-Protein–Coupled Receptors
Source: PLoS Biol. 2009 Aug 18;7(8):e1000172. doi: 10.1371/journal.pbio.1000172 (PMC2718703; doi:10.1371/journal.pbio.1000172)
Supplement: Table S3 — Geometric parameters used in the model. (0.71 MB PDF) [file pbio.1000172.s015.pdf]

Table S3. Geometric parameters used in the model.

**Name**	**Simulation***	**Figure**	**Cell surface (μm^2)/ Cytoplasm volume (μm^3)**	**ICSC surface (μm^2)/ ICSC volume (μm^3)**	**Image**	**Size (μm)**	**Mesh (elements)**
cell 1	C	S9, S11	2826.9/ 14134.5†	1500/ 1413.4	-	-	-
cell 2	C	S10A	2826.9/ 14134.5	500,1000,1500,2000/ 1413.4	-	-	-
cell 3	C	S10A	2826.9/ 14134.5	1500/ 141.3,1413.4,14134.5	-	-	-
cell 4	S	15	-	-	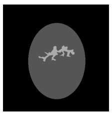	60x60	100x100
cell 5	S	S10B	-	-	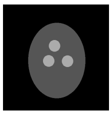	60x60	100x100
cell 6	S	S10B	-	-	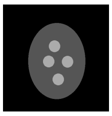	60x60	100x100
cell 7	S	S10B	-	-	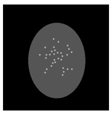	60x60	100x100

* C = compartmental, S = spatial. †equivalent to a sphere of radius 15 μm.
