# Supplementary material for: Persistent cAMP-Signals Triggered by Internalized G-Protein–Coupled Receptors
Source: PLoS Biol. 2009 Aug 18;7(8):e1000172. doi: 10.1371/journal.pbio.1000172 (PMC2718703; doi:10.1371/journal.pbio.1000172)
Supplement: Text S1 — Detailed description of the mathematical model. (0.13 MB PDF) [file pbio.1000172.s016.pdf]

Text S1. Detailed description of the mathematical model.

Simulations were performed in the Virtual Cell modeling environment. A Virtual Cell model is composed of three parts: a so-called physiological model, where cell compartments, molecular species and reactions are defined, one or more applications, where experimental conditions and geometric assumptions are introduced, and one ore more simulations, where the software provides numerical solutions to the model (for a detailed description of the software and of the involved equations see references [1–4]). The physiological model was specified by defining the topological arrangement of the cell compartments (i.e. plasma membrane, cytoplasm and intracellular signaling compartment), and by specifying for each of them the molecular species and mechanisms (reactions and fluxes across membranes) that are involved. Reactions of the type

$$X + A \underset{k_r}{\overset{k_f}{\rightleftarrows}} AX$$

were described according to the mass action law by

$$v = -k_f[X][A] + k_r[AX],$$

where v is the reaction rate, k_f is the forward reaction rate constant, and k_r is the reverse reaction rate constant.

Enzyme-mediated reactions were approximated as irreversible, with Michaelis-Menten rate

$$v = \frac{k_{cat}[E][X]}{K_m + [X]},$$

where [E] is the enzyme concentration, k_{cat} is the catalytic-efficiency constant, and K_m is the Michaelis-Menten constant.

Once the biological model and applications were defined, the software automatically translated them into systems of equations that describe their behavior in time and space.

A first set of simulations was not spatially resolved (compartmental simulations). These are single point simulation based on the physiological model and the geometric assumptions, which are solved using nonlinear ordinary differential equations of the type:

$$\frac{d[X]}{dt} = \sum F_X + \sum v_X,$$

where $\sum F_X$ is the sum of all the inter-compartmental fluxes and $\sum v_X$ is the sum of all the reactions rates v that affect the species X.

Subsequent simulations were spatially resolved (spatial simulations). The spatio-temporal changes to the concentration of molecular species are governed by the mass conservation law, i.e. the rate of change of concentration of a molecular species inside a volume element is the result of all the reactions that affect this species and the diffusion fluxes coming in and out of the element. This physical law is expressed in the Virtual Cell by a set of partial differential equations. If a volume element ΔV, small enough to ignore any spatial changes within it, is arbitrarily selected inside a cell, then the rate of concentration change of the molecular species X inside this volume element is expressed by the following equation:

$$\frac{\partial[X]}{\partial t} = D_X \nabla^2 [X] + \sum v_X,$$

where [X] is the concentration of the species X, D_X is its diffusion coefficient, and $\sum v_X$ is the sum of all the reaction rates v that affect the species X.

For example, the rate of concentration change of cAMP at a given volume element in the cytoplasm is subject to gradient-driven diffusion, degradation by PDEs and sequestration through binding to the four sites presents on the PKA holoenzyme. This is expressed in the model by the following partial differential equation:

$$\frac{\partial[cAMP]}{\partial t} = D_{cAMP}\nabla^2[cAMP] - v(cAMP\_\deg radation) - v(binding\_to\_site1) - v(binding\_to\_site2) - $$
$$- v(binding\_to\_site3) - v(binding\_to\_site4)$$

To simulate a transient ligand application, the ligand was present in the extracellular space at the beginning of the simulation and was removed with kinetics $v = k_p[ligand\_extracellular]$, where $[ligand\_extracellular]$ is the concentration of the ligand in the extracellular compartment and k_p (equal to 0.005 s^{-1} in the simulations that are presented here) is the rate constant of ligand removal.

Since the currently available version of Virtual Cell does not allow to simulate movements of species from one membrane to another, the internalization of the receptor was simulated by considering a first step, equivalent to receptor internalization in endosomes, where the active receptors are transferred from the plasma membrane to the cytoplasm with k_f = 0.005 s^{-1} and k_r = 0. This value for k_f was chosen to match the experimental observation that TSH receptor internalization reaches a maximum after approximately 15-20 min. To simulate an ICSC, the internalized receptors were allowed in a second step to translocate to an intracellular membrane, which contained all the signaling components required for cAMP signaling, with k_f = 2 molecules·μm^{-2}·μM^{-1}·s^{-1} and k_r = 0. This value for k_f was chosen by trying different parameters and selecting the value for which the simulated cAMP traces matched best the FRET data. Ligand internalization was simulated by a flux of ligand from the extracellular compartment to the cytoplasm with reaction rate $k_l[ligand\_extracellular]$, with k_l equal to 0.1 μm·s^{-1}.

The initial concentrations and diffusion coefficients utilized by Neves et al. [5], which are mostly based on experimentally determined values, were used. Initial concentrations, displayed in units of molecules/μm^2 for membrane components and μM for cytosolic components, and values of

diffusion coefficients are provided in Table S1. For those components not shown, the initial concentration was set at zero. Reactions and kinetic parameters are shown in Table S2. Spatial simulations were run using the regular grid, finite volume solver. The mean steady-state concentration of cAMP, obtained by running the model in the absence of ligand until all the components reached steady state, was used as initial concentration for subsequent simulations. The entire model, parameters and geometries are available at http://vcell.org/.

The simulation reported in Figure 15 was carried out assuming the same density of adenylyl cyclase on the cell membrane and the ICSC. The consequences of varying the density of adenylyl cyclase on the ICSC are analyzed in Figure S9. The degree of cAMP signal irreversibility is positively correlated with the density of adenylyl cyclase on the ICSC.

Figure 15 shows the results obtained with a fixed geometry. The effect of varying the shape, size and subcellular location of the ICSC is analyzed in Figure S10. The degree of cAMP signal irreversibility is dependent on the surface area of the ICSC, but not on its volume, as indicated by the results of compartmental simulations (Figure S10A). Figure S10B shows the results obtained by utilizing different ICSC geometries in spatial simulations.

The effect of GPCR recycling on the cAMP response is analyzed in Figure S11. Two possible types of recycling were considered: a short recycling pathway upstream of the ICSC and a long recycling pathway through the ICSC (Figure S11A). Fast recycling through the short pathway causes only minor modifications of the cAMP response (Figure S11B). On the contrary, while the model tolerates slow recycling through the ICSC, fast recycling through this pathway affects the sustained production of cAMP (Figure S11C).

Details of the geometries used in the simulations are provided in Table S3.

1. Loew LM, Schaff JC (2001) The Virtual Cell: a software environment for computational cell biology. Trends Biotechnol 19: 401-406.

2. Smith AE, Slepchenko BM, Schaff JC, Loew LM, Macara IG (2002) Systems analysis of Ran transport. Science 295: 488-491.

3. Slepchenko BM, Schaff JC, Carson JH, Loew LM (2002) Computational cell biology: spatiotemporal simulation of cellular events. Annu Rev Biophys Biomol Struct 31: 423-441.

4. Slepchenko BM, Schaff JC, Macara I, Loew LM (2003) Quantitative cell biology with the Virtual Cell. Trends Cell Biol 13: 570-576.

5. Neves SR, Tsokas P, Sarkar A, Grace EA, Rangamani P, Taubenfeld SM, Alberini CM, Schaff JC, Blitzer RD, Moraru II, Iyengar R (2008) Cell shape and negative links in regulatory motifs together control spatial information flow in signaling networks. Cell 133: 666-680.
